# Corticotropin Stimulation in Adrenal Venous Sampling for Patients With Primary Aldosteronism: The ADOPA Randomized Clinical Trial

**DOI:** 10.1001/jamanetworkopen.2023.38209

**Published:** 2023-10-23

**Authors:** Shumin Yang, Zhipeng Du, Xizi Zhang, Qianna Zhen, Xiaoyu Shu, Jun Yang, Ying Song, Yi Yang, Qifu Li, Jinbo Hu

**Affiliations:** 1 Department of Endocrinology, The First Affiliated Hospital of Chongqing Medical University, Chongqing, China; 2 Department of Endocrinology, Beijing Chao-yang Hospital, Capital Medical University, Beijing, China; 3 Department of Medicine, Monash University, Clayton, Victoria, Australia; 4 Centre for Endocrinology and Metabolism, Hudson Institute of Medical Research, Clayton, Victoria, Australia

## Abstract

**Importance:**

Adrenal venous sampling (AVS) is usually recommended to distinguish between unilateral and bilateral primary aldosteronism (PA) before definitive surgical or medical treatment is offered. Whether a treatment decision based on AVS with or without corticotropin (ACTH) stimulation leads to different biochemical and clinical remission rates in patients with PA remains unclear.

**Objective:**

To evaluate whether treatment decisions based on AVS with or without ACTH stimulation lead to different biochemical and clinical remission rates in patients with PA.

**Design, Setting, and Participants:**

This randomized clinical trial (RCT) was conducted at a tertiary hospital in China from July 8, 2020, to February 20, 2023, among patients with PA aged 18 to 70 years. Patients were followed up for 12 months after the initiation of treatment. An intention-to-diagnose analysis was conducted.

**Interventions:**

Patients were randomly assigned to undergo either ACTH-stimulated or non–ACTH-stimulated AVS.

**Main Outcomes and Measures:**

The primary end point was the proportion of patients with complete biochemical remission after 12 months of follow-up. Secondary outcomes included the proportion of patients who achieved complete clinical remission after 12 months of follow-up, dosages of antihypertensive agents, rate of successful bilateral AVS, and adverse events.

**Results:**

Of 228 patients with PA, 115 were randomized to the non–ACTH-stimulated group (median age, 50.0 years [IQR, 41.0-57.0 years]; 70 males [60.9%]) and 113 to the ACTH-stimulated group (median age, 50.0 years [IQR, 43.5-56.5 years]; 63 males [55.8%]). A total of 68 patients (59.1%) underwent adrenalectomy in the non-ACTH group and 65 (57.5%) in the ACTH group. There was no significant difference in the proportion of patients with complete biochemical remission who were managed on the basis of AVS with vs without ACTH stimulation (with: 56 of 113 [49.6%]; without: 59 of 115 [51.3%]; *P* = .79). There also was no significant difference in the proportion of patients who achieved complete clinical remission between the non-ACTH and ACTH groups (26 of 115 [22.6%] and 31 of 113 [27.4%], respectively; *P* = .40). The intensity of therapy with antihypertensives, successful catheterization of bilateral adrenal veins, and incidence of adverse events did not significantly differ between the non-ACTH and ACTH groups.

**Conclusions and Relevance:**

In this RCT, treatment of PA on the basis of non–ACTH-stimulated or ACTH-stimulated AVS did not lead to significant differences in clinical outcomes for the patients. These results suggest that ACTH stimulation during AVS may not have clinical benefit, at least in the Chinese population.

**Trial Registration:**

ClinicalTrials.gov Identifier: NCT04461535

## Key Points

**Question:**

Does adrenal venous sampling (AVS) with vs without corticotropin (ACTH) stimulation lead to different outcomes in patients with primary aldosteronism?

**Findings:**

In this randomized clinical trial including 228 patients with primary aldosteronism in China, the proportion of patients with complete biochemical remission at follow-up was 51.3% for AVS without and 49.6% for AVS with ACTH stimulation, not a statistically significant difference.

**Meaning:**

The findings suggest that ACTH stimulation during AVS may not have clinical benefit; omitting it could reduce the complexity of the procedure and save costs.

## Teaser

This randomized clinical trial evaluates whether treatment decisions based on adrenal venous sampling conducted with vs without corticotropin (ACTH) stimulation lead to different clinical outcomes among patients with primary aldosteronism.

## Introduction

Primary aldosteronism (PA) is a common form of secondary hypertension.^1,2,3,4^ Unilateral PA (UPA), which is treated by adrenalectomy, and bilateral PA (BPA), which is treated medically, are the 2 major subtypes of PA. Patients with surgically treated PA have better long-term outcomes compared with patients with medically treated PA^5,6,7,8,9,10^; hence, accurate subtyping is important. The current guidelines recommend adrenal venous sampling (AVS) for subtyping in most patients with PA.^11,12,13^

Corticotropin (ACTH) is used during AVS in some centers due to reported benefit of increasing the rates of successful adrenal vein cannulation.^14,15,16,17,18,19,20,21,22,23,24,25,26,27,28^ However, ACTH-stimulated AVS has been reported to decrease the degree of lateralization and potentially mask UPA.^14,16,20,21,22,23,25,27,28^ Studies on clinical outcomes of ACTH are inconsistent, with some studies reporting that postsurgery outcomes were not improved with ACTH use during AVS, while others found that the results of ACTH-stimulated AVS were more useful than nonstimulated AVS in predicting the postoperative outcomes.^22,25,27,28^

To our knowledge, the outcomes of treatment following AVS with or without ACTH stimulation have not been evaluated in a prospective study. Therefore, we conducted a randomized clinical trial (RCT) to evaluate whether the treatment decision (surgical or medical treatment) based on different AVS procedures (with or without ACTH stimulation) would lead to different outcomes in patients with PA. The results of this study may provide insights into the value of ACTH stimulation in AVS for PA subtyping.

## Methods

### Study Design and Participants

This RCT (NCT04461535) was conducted from July 8, 2020, until February 20, 2023, at The First Affiliated Hospital of Chongqing Medical University in Chongqing , China. Beginning July 8, 2020, patients aged 18 to 70 years who were diagnosed with PA in this center were consecutively recruited for the trial. The ethics committee of The First Affiliated Hospital of Chongqing Medical University approved the protocol (available in Supplement 1). Written informed consent was obtained from participants. This study followed the Consolidated Standards of Reporting Trials (CONSORT) reporting guideline (Supplement 1). Inclusion and exclusion criteria are provided in the eMethods in Supplement 2.

### Diagnosis of PA

The PA screening test was considered positive when the plasma aldosterone to renin ratio (ARR) was greater than or equal to 3.16 ng/dL per pg/mL (2.0 ng/dL per mIU/L) (to convert ng/dL of aldosterone to pmol/L, multiply by 27.74, and pg/mL of renin to pmol/L, multiply by 0.0237).^29,30^ Patients who tested positive proceeded to confirmatory testing. Patients who tested negative with the ARR but in whom PA was strongly suspected based on young age (35 years or younger), hypokalemia, or resistant hypertension also proceeded to the confirmatory test. Patients with confirmed PA then underwent adrenal computed tomography (CT) (eMethods in Supplement 2).

### Randomization and Procedures

Patients were randomly assigned to either the ACTH-stimulated or non–ACTH-stimulated group. The randomization method is presented in the eMethods in Supplement 2.

Adrenal venous sampling was performed by 2 experienced endocrinologists (S.Y., Z.D.) between 8 am and 12 pm. Normal saline or ACTH, administered as a continuous infusion, was started 30 minutes before sampling and continued throughout the procedure at 20 mL/h (50 μg/h). Blood samples were collected sequentially from right and left adrenal veins, and blood in the inferior vena cava was collected immediately after the collection of each side of the adrenal vein blood (eMethods in Supplement 2).

Cannulation was considered successful when the selectivity index, namely, the plasma cortisol concentration (PCC) in the adrenal vein divided by the PCC in the inferior vena cava (IVC), was 3 or greater with ACTH stimulation or 2 or greater without ACTH stimulation. The ratio of plasma aldosterone concentration (PAC) to PCC on the side with the higher ratio divided by the contralateral PAC-PCC ratio is defined as the lateralization index (LI). Lateralization of aldosterone excess was defined as an LI of 4 or greater irrespective of ACTH use. Patients with an LI between 2 and 4 together with contralateral suppression (the PAC-PCC ratio of the nondominant side was less than the PAC-PCC ratio of the IVC) or CT showing a typical adenoma on the dominant side were also considered to have lateralized disease. Patients with an LI less than 2 or of 2 to 4 without meeting the above criteria were diagnosed as having BPA.^31,32^ Patients with unilateral disease were referred for adrenalectomy, while those with bilateral disease were treated with mineralocorticoid receptor antagonist (MRA).

In case of technical AVS failure or a bilateral PAC-PCC ratio lower in adrenal venous blood than peripheral blood, adrenalectomy was recommended if the patient met 1 of the following sets of criteria: (1) unilateral nodule (diameter, ≥1 cm) identified on CT, no observable nodules or hyperplasia on the contralateral adrenal gland, and PAC of 20 ng/dL or greater, plasma renin concentration (PRC) less than 3.2 pg/mL, and serum potassium level ≤3.5 mEq/L^33^ (to convert serum potassium to mmol/L, multiply by 1.0) or (2) unilateral nodule (diameter, ≥1 cm) identified on CT, no observable nodules or hyperplasia on the contralateral adrenal gland, and a contralateral index of 0.5 or lower in the adrenal venous sample.^34^

### Outcomes

At follow-up after 1 month and 12 months, blood pressure and medication were recorded and serum potassium, creatinine, PAC, and PRC were measured. Confirmatory tests were performed if the ARR was positive. During follow-up, antihypertensive medication was adjusted by the physicians (Y.S., Q.L.) to achieve a target blood pressure of 140/90 mm Hg or below. For all the patients with PA, except those with UPA who had achieved complete biochemical remission after surgery, at the maximally tolerable dose of MRA, we added conventional antihypertensive agents to reach the target blood pressure if needed.

The primary end point was the proportion of surgically treated patients with complete biochemical remission in the overall cohort after 12 months of follow-up. The secondary outcomes included (1) proportion of surgically treated patients who achieved complete clinical remission in the overall cohort after 12 months of follow-up; (2) daily defined doses (DDDs) of antihypertensive agents (including MRA), blood pressure, and proportion of patients reaching the target blood pressure in each group irrespective of their treatment after 12 months of follow-up; (3) adverse events during the study; and (4) rate of bilaterally successful AVS.

Biochemical and clinical remission were defined based on the Primary Aldosteronism Surgery Outcome criteria.^35^ The DDD is the assumed mean maintenance dose per day for a drug used for its main indication in adults^36^ (eMethods in Supplement 2).

### Statistical Analysis

The trial was designed to have a power of 80% to detect a difference of 19% between the 2 groups in the proportion of complete biochemical remission (eMethods in Supplement 2). A total of 104 patients needed to be enrolled in each group (2-sided α of .05). Taking into account a potential dropout rate of 10%, we aimed to include 115 patients in each group.

Analysis was conducted on an intention-to-diagnose basis: patients were analyzed in the diagnostic group to which they had been randomly assigned, and the patients with failed AVS remained in the group to which they had been randomly assigned, even though their treatment was not determined by AVS.

Sensitivity analysis were conducted (1) excluding patients who were lost to follow-up, (2) excluding patients in whom the treatment decision protocol was violated (if the patient was diagnosed as having UPA but treated with medication or was diagnosed as having BPA but treated with adrenalectomy), (3) excluding patients with failed AVS or with a bilateral PAC-PCC ratio in adrenal venous blood lower than in peripheral blood, (4) excluding patients with an LI of 2 to 4, (5) increasing the AVS selectivity index from 3 to 5 in the ACTH-stimulated group and from 2 to 3 in the non–ACTH-stimulated group, and (6) decreasing the LI from 4 to 2.

The extent of missing data of study variables is shown in eTable 1 in Supplement 2. Methods used to deal with missing data are provided in the eMethods in Supplement 2. Data are expressed as means and SDs or, in the case of skewed distributions, as medians and IQRs. To assess significance of differences between the 2 groups, we used the χ^2^ test or Fisher exact test for categorical data and the unpaired *t* test or Mann-Whitney test for continuous data with and without a normal distribution, respectively. Two-sided *P* < .05 was considered significant. We used IBM SPSS, version 20 (IBM Corp) for statistical analysis.

## Results

### Study Population

**Figure 1.**
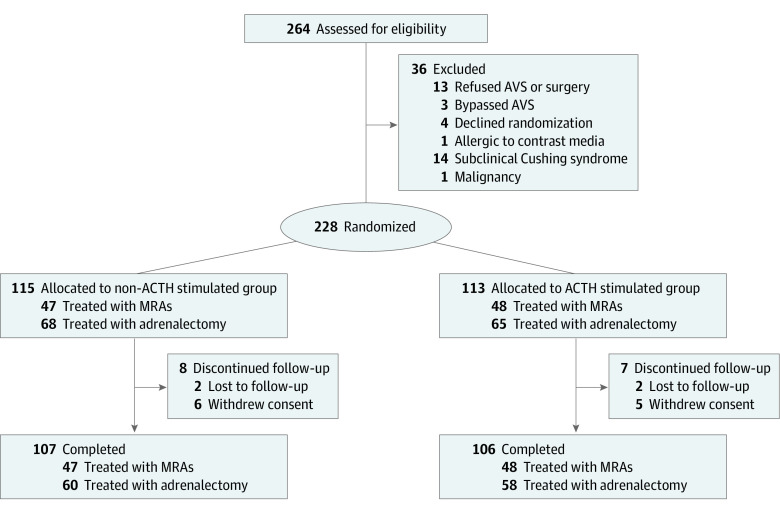
Trial Profile The criteria for bypassing adrenal venous sampling (AVS) were age younger than 35 years and typical characteristics of aldosterone-producing adenoma (plasma aldosterone level >30 ng/dL [to convert to pmol/L, multiply by 27.74], serum potassium level ≤3.5 mEq/L [to convert to mmol/L, multiply by 1.0], and computed tomography indicating unilateral, low-density adenoma with a diameter ≥1 cm). ACTH indicates corticotropin; MRA, mineralocorticoid receptor antagonist.

In total, 228 patients were randomized, of whom 115 were randomized to the non-ACTH group (45 [39.1%], female; 70 [60.9%], male; median age, 50.0 years [IQR, 41.0-57.0 years]) and 113 to the ACTH group (50 [44.2%], female; 63 [55.8%], male; median age, 50.0 years [IQR, 43.5-56.5 years]) (Table 1). In all, 68 patients (59.1%) underwent adrenalectomy in the non-ACTH group and 65 (57.5%) in the ACTH group. At the end of the study, 107 patients in the non-ACTH group (93.0%) and 106 in the ACTH group (93.8%) had completed 12 months of follow-up (Figure 1).

**Table 1.** Baseline Characteristics of All Patients Included in the Final Analysis

Characteristic	Patients (N = 228)^a^	
	Non–ACTH stimulated (n = 115)	ACTH stimulated (n = 113)
Age, y	50.0 (41.0-57.0)	50.0 (43.5-56.5)
Sex, No. (%)		
Female	45 (39.1)	50 (44.2)
Male	70 (60.9)	63 (55.8)
BMI	24.2 (22.8-26.5)	24.9 (22.1-27.2)
Daily defined dose of hypertensive agents^b^	1.3 (1.0-2.0)	1.3 (1.0-2.0)
SBP, mean (SD), mm Hg	149 (18)	152 (17)
DBP, mean (SD), mm Hg	92 (14)	93 (12)
Serum potassium level, mEq/L	3.6 (3.2-3.9)	3.4 (3.1-3.8)
Serum sodium level, mEq/L	142.0 (140.0-144.0)	142.0 (140.0-143.0)
eGFR, mL/min/1.73 m^2^	88.5 (77.9-107.0)	88.2 (76.9-108.0)
Upright PAC, ng/dL	21.8 (15.3-31.8)	23.1 (19.2-34.7)
Upright PRC, pg/mL	1.8 (0.7-4.2)	1.6 (0.6-3.1)
Post-CCT PAC, ng/dL	19.4 (13.6-29.1)	19.7 (15.6-27.8)
Post-CCT PRC, pg/mL	1.9 (0.7-6.6)	2.2 (0.8-5.3)

Abbreviations: ACTH, corticotropin; BMI, body mass index (calculated as weight in kilograms divided by height in meters squared); CCT, captopril challenge test; DBP, diastolic blood pressure; eGFR, estimated glomerular filtration rate; PAC, plasma aldosterone concentration; PRC, plasma renin concentration; SBP, systolic blood pressure.

SI conversion factors: To convert serum potassium and serum sodium to mmol/L, multiply by 1.0; plasma aldosterone to pmol/L, multiply by 27.74; and plasma renin to pmol/L, multiply by 0.0237.

^a^ Data are expressed as the median (IQR) unless otherwise indicated.

^b^ Calculated according to the World Health Organization’s anatomical therapeutic chemical to daily defined dose (ATC/DDD) index.

### AVS Results

There were 106 successful procedures (92.2%) in the non-ACTH group and 105 (92.9%) in the ACTH group. The selectivity index of the left and right adrenal veins was higher in the ACTH group. The median LI was not significantly different between the 2 groups (non-ACTH: 8.06 [IQR, 2.75-23.42]; ACTH: 8.99 [IQR, 1.89-24.89]). When patients who received adrenalectomy and those who received MRA treatment were analyzed separately, results were similar (Table 2).

**Table 2.** Adrenal Vein Sampling Parameters in Non–ACTH-Stimulated and ACTH-Stimulated Groups That Underwent Successful Procedures

Parameter^b^	Total (n = 211)^a^			Adrenalectomy (n = 126)			MRA (n = 85)		
	Non–ACTH stimulated (n = 106)	ACTH stimulated (n = 105)	*P* value	Non–ACTH stimulated (n = 66)	ACTH stimulated (n = 60)	*P* value	Non–ACTH stimulated (n = 40)	ACTH stimulated (n = 45)	*P* value
PAC in left AV, ng/dL	290.7 (111.0-881.9)	2085.0 (525.7-3900.0)	<.001	393.5 (125.5-1272.8)	1915.0 (275.6-5012.9)	<.001	220.1 (103.0-496.6)	2140.0 (1252.5-3475.8)	<.001
PAC in right AV, ng/dL	391.4 (95.2-1313.4)	1636.7 (427.0-5265.0)	<.001	197.3 (65.3-1158.8)	617.5 (288.0-6477.5)	<.001	667.4 (256.7-1949.2)	2090.0 (1105.0-3922.5)	<.001
PCC in left AV, μg/dL	106.5 (49.8-273.0)	617.6 (455.4-935.9)	<.001	122.5 (50.9-262.5)	541.9 (401.1-838.6)	<.001	102.0 (42.7-296.1)	745.7 (569.3-1029.6)	<.001
PCC in right AV, μg/dL	288.2 (129.2-637.3)	894.9 (632.0-1240.2)	<.001	314.9 (139.5-597.4)	885.0 (627.4-1223.6)	<.001	241.7 (90.7-738.0)	942.6 (652.6-1357.7)	<.001
PAC-PCC ratio in left AV, ng/dL per μg/dL	3.31 (0.83-8.83)	2.48 (1.10-5.52)	.32	4.41 (0.83-11.86)	3.59 (0.28-6.62)	.09	2.48 (0.83-4.97)	2.48 (1.66-4.69)	.34
PAC-PCC ratio in right AV, ng/dL per μg/dL	1.66 (0.55-3.59)	1.93 (0.55-4.97)	.48	0.83 (0.28-2.76)	0.83 (0.28-6.62)	.52	2.76 (1.38-3.86)	2.48 (1.38-4.14)	.69
PAC in IVC1, ng/dL	20.4 (13.1-29.9)	41.9 (30.1-59.8)	<.001	25.0 (16.8-34.1)	52.9 (39.4-66.7)	<.001	14.0 (10.6-20.2)	32.7 (24.8-40.5)	<.001
PAC in IVC2, ng/dL	21.7 (15.2-30.4)	39.7 (28.0-56.6)	<.001	26.1 (18.0-36.5)	50.1 (39.2-66.3)	<.001	16.7 (11.4-23.1)	30.8 (23.9-38.4)	<.001
PCC in IVC1, μg/dL	11.4 (8.3-15.4)	23.4 (20.1-25.7)	<.001	12.4 (8.7-14.6)	22.7 (20.0-25.4)	<.001	10.7 (7.1-16.5)	23.8 (20.7-25.9)	<.001
PCC in IVC2, μg/dL	12.7 (9.2-16.8)	21.3 (18.8-23.8)	<.001	13.0 (9.8-16.3)	20.8 (18.2-22.8)	<.001	12.3 (7.7-18.2)	21.5 (19.4-25.3)	<.001
PAC-PCC ratio in IVC1, ng/dL per μg/dL	1.93 (1.10-3.31)	1.93 (1.10-2.76)	.65	2.21 (1.38-3.31)	2.21 (1.66-3.31)	.42	1.38 (1.10-1.93)	1.38 (1.10-1.93)	.77
PAC-PCC ratio in IVC2, ng/dL per μg/dL	1.66 (1.10-3.03)	1.93 (1.38-2.76)	.36	2.21 (1.38-3.31)	2.48 (1.93-3.03)	.20	1.38 (0.83-1.93)	1.38 (1.10-1.93)	.63
Lateralization index	8.06 (2.75-23.42)	8.99 (1.89-24.89)	.48	16.80 (8.08-36.03)	17.93 (10.97-41.16)	.65	2.20 (1.42-3.63)	1.70 (1.22-2.52)	.13
Selectivity index in left AV	11.46 (4.86-20.39)	30.13 (20.44-39.73)	<.001	12.00 (4.97-20.56)	25.47 (18.20-38.80)	<.001	9.13 (4.71-19.96)	34.98 (26.38-40.98)	<.001
Selectivity index in right AV	26.04 (11.91-43.79)	42.25 (31.82-64.30)	<.001	26.91 (13.27-43.79)	46.26 (32.17-63.12)	<.001	21.22 (9.74-45.48)	39.97 (31.28-66.34)	.002

Abbreviations: ACTH, corticotropin; AV, adrenal vein; IVC, inferior vena cava; IVC1, inferior vena cava blood collected immediately after the collection of left adrenal vein blood; IVC2, inferior vena cava blood collected immediately after the collection of right adrenal vein blood; MRA, mineralocorticoid receptor antagonist; PAC, plasma aldosterone concentration; PCC, plasma cortisol concentration.

SI conversion factors: To convert plasma aldosterone to pmol/L, multiply by 27.74; plasma renin to pmol/L, multiply by 0.0237; and plasma cortisol to nmol/L, multiply by 27.588.

^a^ Patients with failed AVS were excluded (9 in the non–ACTH-stimulated and 8 in the ACTH-stimulated group).

^b^ Data are expressed as the median (IQR).

In the non-ACTH group, 9 of 115 patients (7.8%) had failed cannulation (5 [55.6%] of the right and 4 [44.4%] of the left adrenal vein). Of the 106 successful procedures, 70 (66.0%) showed lateralization of aldosterone production (64 [91.4%] with an LI > 4 and 6 [8.6%] with an LI of 2-4 plus contralateral suppression), 31 (29.2%) showed no lateralization, and 5 (4.7%) were considered inconclusive due to a lower PAC-PCC ratio in bilateral adrenal veins compared with the peripheral vein. All 31 patients who were diagnosed with BPA by AVS were treated with MRA, while 65 of the 70 who were diagnosed with UPA by AVS (92.9%) underwent adrenalectomy. Five of the patients with UPA (7.1%) declined surgery and chose MRA treatment. One of the 5 patients with inconclusive AVS results (20.0%) met the criteria for surgery according to the prespecified study protocol and underwent adrenalectomy, and the other 4 (80.0%) were treated with MRA. Among the 9 patients with unsuccessful AVS procedures, 1 (11.1%) met criteria for surgery, 1 (11.1%) requested surgery by herself and underwent adrenalectomy, and the other 7 (77.8%) were treated with MRA.

In the ACTH group, 8 of the 113 procedures (7.1%) were unsuccessful due to failed cannulation of the right adrenal vein. In the 105 successful procedures, 64 (61.0%) showed lateralization of aldosterone production (60 [93.8%] had an LI > 4, 2 [3.1%] had an LI of 2-4 plus contralateral suppression, and 2 [3.1%] had an LI of 2-4 plus adrenal adenoma on CT), 39 (37.1%) showed no lateralization, and 2 (1.9%) were considered inconclusive due to a lower PAC-PCC ratio in the bilateral adrenal veins than the peripheral vein. All 39 patients who were diagnosed with BPA by AVS were treated with MRA, and 59 of the 64 who were diagnosed with UPA by AVS underwent adrenalectomy (92.2%), while 5 patients (7.8%) with UPA chose MRA treatment. One of the 2 patients (50.0%) with inconclusive AVS results met the criteria for surgery according to prespecified study protocol and underwent adrenalectomy, while the other was treated with MRA. Among the 8 patients with unsuccessful AVS procedures, 4 (50.0%) met the criteria for surgery, whereas 1 (12.5%) requested surgery and hence underwent adrenalectomy and 3 (37.5%) were treated with MRA.

### Outcomes

**Figure 2.**
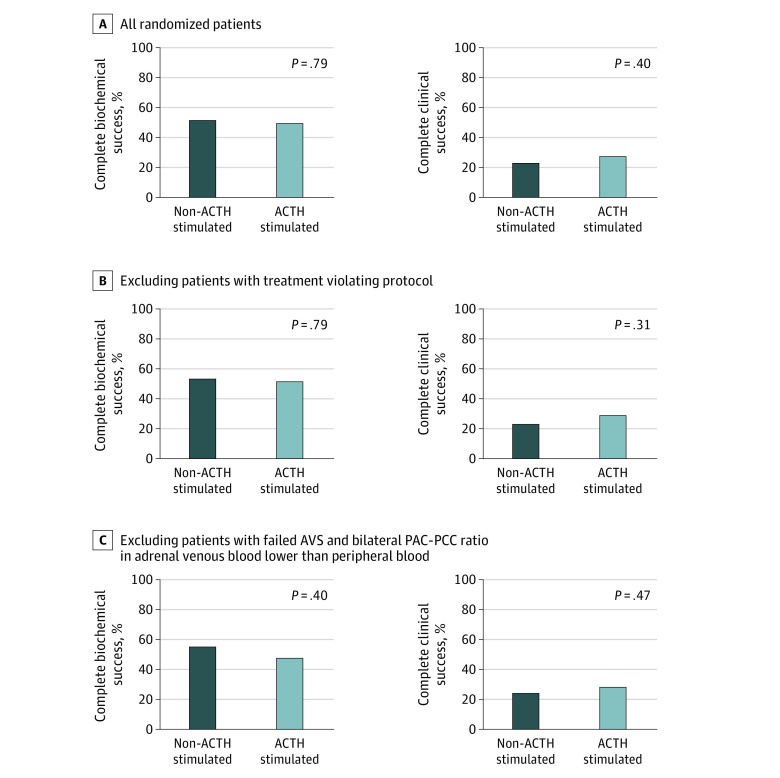
Proportion of Patients With Complete Biochemical and Clinical Remission AVS indicates adrenal venous sampling; PAC, plasma aldosterone concentration; and PCC, plasma cortisol concentration.

The data collected at follow-up visits used to calculate the outcomes are shown in eTable 2 in Supplement 2. For the primary end point, among the patients who achieved complete biochemical success, there was no significant difference in the proportion of those receiving ACTH-stimulated AVS (56 of 113 [49.6%]) or unstimulated AVS (59 of 115 [51.3%]) (*P* = .79) (Figure 2A). The proportion of patients with complete biochemical remission among those treated surgically was also analyzed, and there was no significant difference between the non-ACTH group and the ACTH group (59 of 68 [86.8%] vs 56 of 65 [86.2%], respectively; *P* = .92). Three of 228 patients (1.3%) (2 of 113 [1.8%] in the ACTH group and 1 of 115 [0.9%] in the non-ACTH group) showed partial biochemical remission after adrenalectomy. Although hypokalemia was completely resolved, they all had persistent postoperative hyperaldosteronism (eTable 3 in Supplement 2).

For the secondary end points, the proportion of patients who underwent adrenalectomy and achieved complete clinical success was not significantly different between the non-ACTH and ACTH groups (26 of 115 [22.6%] and 31 of 113 [27.4%], respectively; *P* = .40) (Figure 2A). The DDDs of antihypertensive agents (including MRA), blood pressure, and the proportion of patients reaching target blood pressure at final assessment were also not significantly different between the 2 groups (Table 3).

**Table 3.** Secondary Outcomes of Patients After 12 Months of Follow-Up^a^

Outcome	Total (N = 228)			Adrenalectomy (n = 133)			MRA (n = 95)		
	Non–ACTH stimulated (n = 115)	ACTH stimulated (n = 113)	*P* value^b^	Non–ACTH stimulated (n = 68)	ACTH stimulated (n = 65)	*P* value^b^	Non–ACTH stimulated (n = 47)	ACTH stimulated (n = 48)	*P* value^b^
Defined daily dose, median (IQR)	1.0 (0.0-1.7)	0.6 (0.0-2.0)	.35	0 (0.0-1.0)	0 (0.0-0.1)	.10	1.6 (1.0-2.3)	1.5 (0.8-2.3)	.75
SBP, mean (SD), mm Hg	128 (13)	131 (18)	.26	127 (14)	129 (19)	.62	130 (13)	133 (16)	.25
DBP, mean (SD), mm Hg	85 (10)	87 (13)	.24	85 (11)	87 (14)	.34	85 (10)	87 (12)	.50
Patients achieving target blood pressure, No. (%)	78 (67.8)	69 (61.1)	.29	47 (69.1)	42 (64.6)	.58	31 (66.0)	27 (56.3)	.33

Abbreviations: ACTH, corticotropin; DBP, diastolic blood pressure; MRA, mineralocorticoid receptor antagonist; SBP, systolic blood pressure.

^a^ Data are expressed as the median (IQR) unless otherwise indicated.

^b^ Comparison between non–ACTH-stimulated and ACTH-stimulated groups.

During the study, 80 adverse events were reported in 228 patients, of which 6 (7.5%) were serious adverse events (eTable 4 in Supplement 2). The number of patients experiencing adverse events or serious adverse events did not differ significantly between the 2 groups.

### Sensitivity Analysis

After excluding patients who were lost to follow-up in the study (eTable 5 in Supplement 2), there were no clinically relevant differences in terms of the primary and secondary end points between the 2 groups (eTable 6 in Supplement 2). After excluding patients whose treatment violated the protocol (eTable 7 in Supplement 2) or those who failed AVS plus those with a bilateral PAC-PCC ratio in adrenal venous blood lower than in peripheral blood (eTable 8 in Supplement 2), there were no clinically relevant differences in terms of the primary or secondary end points between the non-ACTH and the ACTH groups (Figure 2B and C and eTables 9 and 10 in Supplement 2).

There were 17 patients (14.8%) in the non-ACTH group and 16 patients (14.2%) in the ACTH group with an LI between 2 and 4. After they were excluded, the results of the analysis were similar to those observed when those patients were not excluded (eTables 11 and 12 in Supplement 2). Increasing the AVS selectivity index (eTable 13 in Supplement 2) or decreasing the LI (eTable 14 in Supplement 2) did not change the study outcomes.

## Discussion

In this RCT, we found that the treatment decision based on AVS with or without ACTH stimulation did not result in clinically significant differences in patient outcomes. Currently, there is marked variation in the conduct of AVS across different centers worldwide. The use of ACTH stimulation during AVS has generated much discussion, but there is no consensus on its use. The findings of this study offer valuable insights into the value of ACTH stimulation during AVS.

The key finding of our study is that a similar proportion of surgically treated patients achieved complete biochemical success, irrespective of ACTH use during their AVS. Previous studies have mostly reported a decrease in the median LI following ACTH stimulation when compared with the LI prior to ACTH.^14,16,20,21,22,23,25,27,28^ In a study by Wannachalee et al,^37^ 24% of patients had discordant AVS results when comparing their lateralization status before and after ACTH stimulation. A patient may be considered to have UPA based on pre-ACTH AVS but BPA based on post-ACTH AVS results, or vice versa. Chee et al^14^ reported cases of failed surgery in patients with an elevated LI before ACTH who experienced loss of lateralization after ACTH. However, the accuracy of the treatment decision is not always known if patients with discordant AVS results are not referred for surgery. Hence, our study is unique in evaluating the final surgical outcome.

Our finding that clinical outcomes were not different between ACTH-stimulated and non–ACTH-stimulated groups is different than in previous reports.^22,25,27,28,38^ Yatabe et al^27^ found that the ACTH-stimulated LI correlated better with postoperative clinical outcomes than the non–ACTH-stimulated LI and concluded that post-ACTH AVS indexes may be more useful for predicting treatment outcome after unilateral adrenalectomy. Kobayashi et al^38^ conducted a multicenter retrospective study on 314 patients with PA with both basal and ACTH-stimulated AVS data who underwent adrenalectomy and found that the use of data only from unstimulated AVS might lead to worse surgical outcomes than the use of ACTH-stimulated AVS results. A possible reason for the relatively higher rates of biochemical and clinical success in our study is the high proportion of patients with florid UPA in the study. The LI reported in patients deemed to have UPA was much higher than the threshold of 4 and may be less likely to fall below the threshold before or after ACTH stimulation. Furthermore, a large proportion of patients with UPA likely has *KCNJ5* sequence variations, the most common sequence variation in aldosterone-producing adenomas in China and Japan,^39,40,41^ and these patients tend to achieve biochemical and clinical cure.^42,43^

The finding that ACTH stimulation during AVS increased the selectivity index in bilateral adrenal veins is consistent with previous reports.^14,15,16,17,18,19,20,21,22,23,24,25,26,27,28^ However, ACTH stimulation in our study did not result in a higher rate of successful adrenal vein catheterization. Monticone et al^19^ reported that cannulation success after ACTH was 87% compared with 49% obtained in basal conditions; therefore, the authors suggested centers with low success rates under basal conditions should consider performing AVS after ACTH stimulation. In our study, successful catheterization in bilateral adrenal veins in the non-ACTH and ACTH groups was similarly high at 92.2% and 92.9%, respectively, most likely due to the dedicated expertise of AVS operators at our study center. This was similarly demonstrated in the study by Chee et al,^14^ in which ACTH use improved cannulation success only in the center with less-experienced operators, while the benefit was minimal in the center with experienced operators.

### Strengths and Limitations

This study has strengths, which overall lie in its randomized design and selection of an objective patient outcome as the primary end point. Additionally, the study population included not only patients with UPA but also BPA, in contrast to most previous retrospective studies focusing on unilateral disease. We also performed the AVS procedures according to accepted protocols with a high rate of cannulation success.

This study also has limitations. The main limitation was that the patients’ response to ACTH could not be fully evaluated in that they either had ACTH or did not have ACTH. It is not known how many patients would have had discordant AVS results, which is the main group that causes uncertainty. If we had performed AVS both before and after ACTH stimulation and then made the treatment decision based on one or the other result (randomized), we would have even greater insight into the effect of ACTH stimulation on AVS outcomes and treatment decisions. Based on previous studies, ACTH stimulation may increase the rate of successful cannulation, so our study was designed as a superiority trial, but the actual rate of successful cannulation was higher than 92% in both groups, and we did not find that ACTH-stimulated AVS was superior to non–ACTH-stimulated AVS, which may lead to bias toward the null in superiority trials. Therefore, it may be necessary to conduct a noninferiority trial in the future to prove that non–ACTH-stimulated AVS is not inferior to ACTH-stimulated AVS. In addition, only Chinese patients were included in our study, and the generalizability of our results to other racial and ethnic groups requires further study.

## Conclusions

In this RCT, treatment of PA on the basis of non–ACTH-stimulated or ACTH-stimulated AVS did not lead to significant differences in biochemical and clinical outcomes for the patients. In the diagnostic workup of patients with PA, ACTH infusion during AVS adds another layer of complexity to an already challenging procedure. Our results suggest that ACTH-stimulated AVS does not offer additional benefit compared with nonstimulated AVS, at least in the Chinese population.

## Notes

Supplement 1.
Trial Protocol

Supplement 2. **eMethods.** Supplemental Methods **eTable 1.** Proportion of Missing Data in Study Variables **eTable 2.** Outcomes of Patients in the Trial at 1st and 12th-Month Follow-Up **eTable 3.** Persistent Hyperaldosteronism in Patients Who Underwent Adrenalectomy **eTable 4.** Adverse and Serious Adverse Events **eTable 5.** Baseline Characteristics of the Patients Who Completed Follow-Up **eTable 6.** Outcomes at 12th-Month Follow-Up in Patients Who Completed Follow-Up **eTable 7.** Baseline Characteristics of the Patients Excluding Those Whose Treatment Violated the Protocol **eTable 8.** Baseline Characteristics of the Patients Excluding Those With Failed AVS Plus Bilateral PAC-PCC Ratio in Adrenal Venous Blood Lower Than Peripheral Blood **eTable 9.** Secondary Outcomes at 12th-Month Follow-Up in Patients Excluding Those Whose Treatment Violated the Protocol **eTable 10.** Secondary Outcomes at 12th-Month Follow-Up in Patients Excluding Those With Failed AVS Plus Bilateral PAC-PCC Ratio in Adrenal Venous Blood Lower Than Peripheral Blood **eTable 11.** Baseline Characteristics of the Patients Excluding Those With LI 2 to 4 **eTable 12.** Outcomes at 12th-Month Follow-Up in Patients Excluding Those With LI 2 to 4 **eTable 13.** Outcomes at 12th-Month Follow-Up With Increased Selectivity Index **eTable 14.** Outcomes at 12th-Month Follow-Up With Decreased LI
eReferences

Supplement 3. **Nonauthor Collaborators.** Chongqing Primary Aldosteronism Study (CONPASS) Group

Supplement 4.
Data Sharing Statement
