# Supplementary material for: Corticotropin Stimulation in Adrenal Venous Sampling for Patients With Primary Aldosteronism: The ADOPA Randomized Clinical Trial
Source: JAMA Netw Open. 2023 Oct 23;6(10):e2338209. doi: 10.1001/jamanetworkopen.2023.38209 (PMC10594148; doi:10.1001/jamanetworkopen.2023.38209)
Supplement: Supplement 1. — Trial Protocol [file jamanetwopen-e2338209-s001.pdf]

Corticotropin Stimulation in Adrenal Venous Sampling for Patients With Primary Aldosteronism: The ADOPA Randomized Clinical Trial

Supplement 1

This supplement contains the following items:

1. Original protocol in Chinese, final protocol in Chinese, original protocol in English and final protocol in English.

2. Statistical analysis plan in Chinese and in English.

3. Patient informed consent in Chinese and in English.

4. Baseline case report form (CRF) and Follow-up CRF.

5. Adrenal vein sampling (AVS) data collecting form.

Original protocol in Chinese 3

Final protocol in Chinese 9

Original protocol in English 15

Final protocol in English 22

Statistical analysis plan in Chinese 30

Statistical analysis plan in English 31

Patient informed consent in Chinese 34

Patient informed consent in English 37

Baseline case report form (CRF) 41

Follow-up CRF 44

Adrenal vein sampling (AVS) data collecting form 48

Original protocol in Chinese

ACTH 兴奋和非 ACTH 兴奋的肾上腺静脉取血对原发性醛固酮增多症患者的影响：一项基于临床结局的随机、双盲研究

一、研究背景

原发性醛固酮增多症(Primary Aldosteronism, PA)是最常见的继发性高血压之一，占所有高血压患者的 5%-10%[1-3]。该病是由于肾上腺皮质病变导致醛固酮过度分泌，患者以高血压、低血钾、低肾素及高醛固酮为主要临床特征。PA 患者肾上腺病变包括单侧腺瘤、单侧醛固酮癌、双侧肾上腺增生等，其中单侧醛固酮瘤(aldosterone producing adenoma，APA)和双侧肾上腺皮质增生 (bilateral adrenocortical hyperplasia，BAH)是 PA 最常见的两种类型[4]。对于单侧醛固酮瘤，建议手术治疗，多数 APA 患者通过手术治疗可以达到根治目的；对于双侧肾上腺皮质增生，则采用口服盐皮质激素受体拮抗剂（如螺内酯）治疗。我们及其他团队的研究表明，手术患者的远期预后（比如肾功能的下降，心脑血管事件等风险）较口服药物治疗患者的预后更好[5-6]。由此可见，明确 PA 患者是单侧醛固酮瘤还是双侧肾上腺皮质增生对于患者的预后至关重要。

目前，PA 的诊断分为筛查、确诊及分型三个步骤。国内外指南建议对大部分高血压患者进行 PA 的筛查，包括持续性收缩压>150mmHg 或舒张压>100mmHg、伴有低血钾、伴肾上腺意外瘤的高血压患者等[7-10]。首先通过检测患者醛固酮、肾素并计算醛固酮/肾素比值进行筛查，筛查阳性的患者进一步通过确诊试验（如卡托普利抑制试验、静脉盐水负荷试验等）明确诊断，对于诊断为 PA 的患者，需要进一步分型明确是单侧还是双侧肾上腺病变，以决定是否手术治疗。分型诊断的方法主要包括肾上腺 CT 和肾上腺静脉取血术(AVS)，由于 CT 的准确性只有 70-90%[11-13]，国内外指南均建议，除部分符合典型的 APA 影像学及临床特征（年龄小于 35 岁，伴低血钾病史，且 CT 可见典型的单侧肾上腺腺瘤）的 PA 患者外，其余有手术意愿的 PA 患者均需进一步行 AVS 明确分型，判断醛固酮优势分泌侧，为手术治疗提供依据[7-10]。

AVS 目前主要分为常规 AVS 和促肾上腺皮质激素(ACTH)兴奋的 AVS。肾上腺静脉短而细，且存在解剖变异，此外，肾上腺激素分泌有波动性，这些因素都可能影响 AVS 的成功率，目前国内外报道的 AVS 的成功率为 60-95%[14-16]。由于肾上腺激素存在脉冲式分泌的特点，若取血时激素分泌处于低谷期可能影响结果判断；此外，AVS 除了检测醛固酮还需要检测皮质醇，手术应激对皮质醇有较大影响，这也可能影响结果判断，而 ACTH 刺激可使得肾上腺醛固酮和皮质醇分泌显著增多，有研究显示 ACTH 兴奋可减少激素波动，提高 AVS 成功率[17-18]。课题组前期的回顾性研究也发现，ACTH 兴奋可提高 AVS 成功率，且不影响对肾上腺病变侧的判定[19-20]。但也有研究报道，ACTH 兴奋可能将 PA 患者中单侧肾上腺病变误诊为双侧肾上腺病变，使得患者错失手术机会[21-22]。

综上，ACTH 兴奋的 AVS 可能提高肾上腺静脉插管成功率，但也可能会影响肾上腺醛固酮病变侧的判断。目前，国内外对于常规 AVS 和 ACTH 兴奋的 AVS 对 PA 患者的远期预后的影

响尚未见报道。因此，课题组拟进行一项前瞻性、随机试验，比较基于 ACTH 兴奋的 AVS 和常规的 AVS（即非 ACTH 兴奋的 AVS）判断 PA 患者的醛固酮优势分泌，并确定临床治疗策略，比较 PA 患者的远期预后，从而为临床推荐 AVS 手术方式提供证据。

参考文献

[1] Rossi GP, Bernini G, Caliumi C, et al. A prospective study of the prevalence of primary aldosteronism in 1,125 hypertensive patients[J]. J Am Coll Cardiol 2006; 48(11): 2293-300.

[2] Monticone S, Burrello J, Tizzani D, et al. Prevalence and Clinical Manifestations of Primary Aldosteronism Encountered in Primary Care Practice[J]. Journal of the American College of Cardiology 2017; 69(14): 1811-20.

[3] Xu Zhixin,Yang Jun,Hu Jinbo et al. Primary Aldosteronism in Patients in China With Recently Detected Hypertension.[J] .J Am Coll Cardiol, 2020, 75: 1913-1922.

[4] Mulatero P, Sechi LA, Williams TA, et al. Subtype diagnosis, treatment, complications and outcomes of primary aldosteronism and future direction of research: a position statement and consensus of the Working Group on Endocrine Hypertension of the European Society of Hypertension [J]. Journal of Hypertension, 2020, 38(10): 1929-1936.

[5] Monticone S, D'Ascenzo F, Moretti C, et al. Cardiovascular events and target organ damage in primary aldosteronism compared with essential hypertension: a systematic review and meta-analysis[J]. The Lancet Diabetes & Endocrinology. 2018, 6(1):41-50.

[6] Hundemer Gregory L,Curhan Gary C,Yozamp Nicholas et al. Renal Outcomes in Medically and Surgically Treated Primary Aldosteronism.[J] .Hypertension, 2018, 72: 658-666.

[7] Funder JW, Carey RM, Mantero F, et al. The Management of Primary Aldosteronism: Case Detection, Diagnosis, and Treatment: An Endocrine Society Clinical Practice Guideline[J]. J Clin Endocrinol Metab, 2016, 101(5): 1889-1916.

[8] 原发性醛固酮增多症诊断治疗的专家共识[J]. 中华内分泌代谢杂志, 2016, 32(03): 188-195.

[9] Amar Laurence,Baguet Jean Philippe,Bardet Stéphane et al. SFE/SFHTA/AFCE primary aldosteronism consensus: Introduction and handbook.[J] .Ann Endocrinol (Paris), 2016, 77: 179-86.

[10] Nishikawa T, Omura M, Satoh F, et al. Guidelines for the diagnosis and treatment of primary aldosteronism--the Japan Endocrine Society 2009. Endocrine journal 2011; 58(9): 711-21.

[11] Young William F,Stanson Anthony W,Thompson Geoffrey B et al. Role for adrenal venous sampling in primary aldosteronism.[J] .Surgery, 2004, 136: 1227-35.

[12] Rossi Gian Paolo,Rossitto Giacomo,Amar Laurence et al. Clinical Outcomes of 1625 Patients With Primary Aldosteronism Subtyped With Adrenal Vein Sampling[J] .Hypertension, 2019, 74: 800-808.

[13] Kempers Marlies J E,Lenders Jacques W M,van Outheusden Lieke et al.

Systematic review: diagnostic procedures to differentiate unilateral from bilateral adrenal abnormality in primary aldosteronism.[J] .Ann Intern Med, 2009, 151: 329-37.

[14] Husainy Mohammad Ali,Fang Cheng,Nicolescu Ana et al. Success in adrenal venous sampling between two protocols: experience at a tertiary centre.[J] .J Clin Pathol, 2017, 70: 91-92.

[15] Rossitto Giacomo,Battistel Michele,Barbiero Giulio et al. The subtyping of primary aldosteronism by adrenal vein sampling: sequential blood sampling causes factitious lateralization.[J] .J Hypertens, 2018, 36: 335-343.

[16] Williams TA, Lenders JWM, Mulatero P, et al. Outcomes after adrenalectomy for unilateral primary aldosteronism: an international consensus on outcome measures and analysis of remission rates in an international cohort[J]. Lancet Diabetes Endocrinol. 2017;5(9):689 - 699.

[17] Elliott Panda,Holmes Daniel T,Adrenal vein sampling: substantial need for technical improvement at regional referral centres.[J] .Clin Biochem, 2013, 46: 1399-404.

[18] Monticone Silvia,Satoh Fumitoshi,Giacchetti Gilberta et al. Effect of adrenocorticotropic hormone stimulation during adrenal vein sampling in primary aldosteronism.[J] .Hypertension, 2012, 59: 840-6.

[19] 杜志鹏,程庆丰,宋颖,等.探讨促肾上腺皮质激素兴奋在肾上腺静脉取血中的应用价值[J].中华内分泌代谢杂志,2021,37(02):129-134..

[20] Laurent Irakoze,Astère Manirakiza,Zheng Fengfan et al. Adrenal venous sampling with or without adrenocorticotropic hormone stimulation: A meta-analysis.[J] .J Clin Endocrinol Metab, 2018, undefined: undefined.

[21] Seccia, T.M.; Miotto, D.; de Toni, et al. Adrenocorticotropic hormone stimulation during adrenal vein sampling for identifying surgically curable subtypes of primary aldosteronism: Comparison of 3 different protocols[J]. Hypertension 2009, 53, 761 - 766.

[22] El Ghorayeb Nada,Mazzuco Tânia L,Bourdeau Isabelle et al. Basal and Post-ACTH Aldosterone and Its Ratios Are Useful During Adrenal Vein Sampling in Primary Aldosteronism.[J] .J Clin Endocrinol Metab, 2016, 101: 1826-35.

二、研究概述

ACTH 兴奋可提高 AVS 插管成功率，但可能影响优势侧判断的准确性，但目前国内外尚无 RCT 研究比较 ACTH 兴奋 AVS 和普通 AVS 对 PA 患者预后的影响。本研究旨在评价基于 ACTH 兴奋 AVS 或普通 AVS，判断 PA 患者有无醛固酮单侧优势分泌，进而确定临床治疗方案，即手术治疗或药物治疗，并通过治疗后随访，比较两组患者的临床预后。终点事件包括完全生化缓解率、高血压缓解情况、生活质量评分及不良事件等。

三、研究设计

（一）研究描述

本研究为随机、双盲（患者及 AVS 操作者）、对照试验，基于普通 AVS（对照组）或 ACTH 兴奋 AVS，判断 PA 患者有无醛固酮单侧优势分泌，进而确定临床治疗方案，即手术治疗或药物治疗，并长期随访临床转归；确证基于 ACTH 兴奋 AVS 的 PA 患者组预后是否优于基于普通 AVS 组，从而为临床 AVS 的方法选择提供证据。

（二）流程图

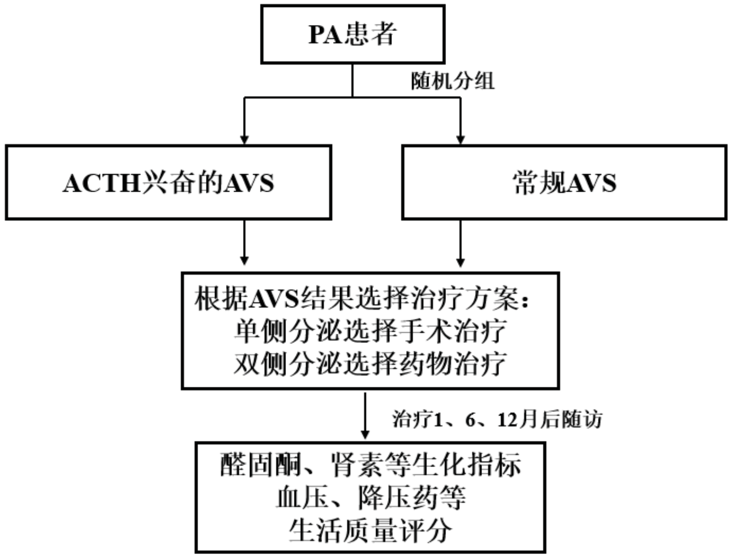

图 1.研究流程图

（三）纳排标准

1、纳入标准

符合下列条件者方可成为本研究的受试者:

◆ 了解试验全过程，自愿接受随机化分组、干预及随访；

◆ 自愿参加该研究并签署知情同意书，愿意按要求完成所有随访；

◆ 年龄 18 岁以上，男女不限，有法定行为能力；

◆ PA 筛查阳性（ARR>10）且至少两个 PA 确诊试验（CCT、SIT 或 FST）为阳性的高血压患者；

◆ 在确定醛固酮优势侧分泌情况下，有明确意愿同意接受肾上腺单侧切除术。

2、排除标准

有以下情况之一不能纳入本研究：

◆ 孕妇或哺乳期妇女

◆ 有恶性肿瘤病史者

◆ 不适合或拒绝接受 AVS 或单侧肾上腺切除手术，或对 ACTH 过敏；

◆ PA 合并库欣综合征（包括亚临床库欣）；

◆ 考虑糖皮质激素可抑制性醛固酮增多症(GRA)或肾上腺皮质癌；

◆ 现已服用药物，且无法停药或换药，而该药物与本研究规定的治疗方法有相关作用，

或者会导致高血压血压增高，例如糖皮质激素等。

◆ 基线时合并严重心脏病（包括起搏器植入术后）、严重心功能不全、严重贫血(Hb<60g/L)、3 月内发生过脑卒中或急性冠脉综合征，严重的腹水及肝硬化等，严重干扰 PA 后续治疗或与健康相关的生活质量评估等。

◆ 酗酒、吸毒病人，以及有精神障碍不能合作者

3、剔除标准

如果患者出现以下情况之一，则应考虑退出试验：

◆ 患者未按照研究标准选择治疗方式或失访；

◆ 发生严重 CVD，如心肌梗死、卒中和严重心功能不全；

◆ 发生严重肾功能不全。

（四）随机对照试验

1、患者来源

有手术意愿的原发性醛固酮增多症患者。

2、受试者分组及随机的实施：

利用 SPSS 软件中 Rv.Uniform 函数产生的随机数字表并随机分为 ACTH 组及非 ACTH 组，以此确定患者的随机号和组别。

3、干预措施

（1）普通 AVS 组：AVS 插管后分别采集右侧和左侧肾上腺静脉血以及相应的外周静脉血，肾上腺皮质醇 / 外周静脉皮质醇（即选择指数，selectivity index，SI）≥2 判定为插管成功，两侧经肾上腺静脉皮质醇校正过的醛固酮比值(高侧肾上腺静脉血醛固酮 / 肾上腺皮质醇与低侧肾上腺静脉血醛固酮 / 肾上腺皮质醇的比值,即优势侧指数,(lateralization index，LI)≥4，建议行单侧肾上腺切除术。

（2）ACTH 兴奋 AVS 组：术前微量泵入 ACTH（250μg 即 25IU/支，100ml 5-10%葡萄糖配制，50μg/h 即 20ml/h)，ACTH 静滴 30min 后，分别采集右侧和左侧肾上腺静脉血以及相应的外周静脉血。SI≥3 认为插管成功；LI≥4 建议行单侧肾上腺切除术。

4、资料收集及随访

基线和治疗后第 1、6 月及 12 月收集患者血标本，送检血常规、肾功、电解质、PAC 和 PRC，必要时重复确诊试验，记录血压及用药情况，测量身高、体重，记录生活质量评分（表 1）

表 1. 随访流程图

参数	基线	1 月 (V1)	6 月 (V2)	12 月 (V3)
**基本资料**				
人口学资料	√			
疾病史	√			√
**临床资料**				
身高	√			√

体重	√			√
腰围	√			√
血压	√	√	√	√
降压药物	√	√	√	√
**血标本**				
电解质	√	√	√	√
肾功 2 号	√	√	√	√
血常规	√	√	√	√
PAC/PRC	√	√	√	√
PA 确诊试验	√	?		?
生活质量评分	√			√

备注：? 表示必要时

4、终点指标

（1）主要终点

- 随访 12 个月时，完全生化缓解的比例。

（2）次要终点

- 随访 12 个月时，临床缓解的比例。
- 随访 12 个月时，血压降至 140/90mmHg 时，两组间降压药物 DDD 值 (Daily Defined Doses, DDD)。
- 两组 AVS 插管成功率
- 干预后 12 个月时，两组间健康相关生活质量评分。
- 两组间不良事件比较，包括肾上腺静脉出血及相关的肾上腺皮质功能减退、高血压亚急症、过敏性休克、静脉血栓、肺栓塞。

Final protocol in Chinese

ACTH 兴奋和非 ACTH 兴奋的肾上腺静脉取血对原发性醛固酮增多症患者的影响：一项基于临床结局的随机研究

一、研究背景

原发性醛固酮增多症(Primary Aldosteronism, PA)是最常见的继发性高血压之一，占所有高血压患者的 5%-10%[1-3]。该病是由于肾上腺皮质病变导致醛固酮过度分泌，患者以高血压、低血钾、低肾素及高醛固酮为主要临床特征。PA 患者肾上腺病变包括单侧腺瘤、单侧醛固酮癌、双侧肾上腺增生等，其中单侧醛固酮瘤(aldosterone producing adenoma，APA)和双侧肾上腺皮质增生 (bilateral adrenocortical hyperplasia，BAH)是 PA 最常见的两种类型[4]。对于单侧醛固酮瘤，建议手术治疗，多数 APA 患者通过手术治疗可以达到根治目的；对于双侧肾上腺皮质增生，则采用口服盐皮质激素受体拮抗剂（如螺内酯）治疗。我们及其他团队的研究表明，手术患者的远期预后（比如肾功能的下降，心脑血管事件等风险）较口服药物治疗患者的预后更好[5-6]。由此可见，明确 PA 患者是单侧醛固酮瘤还是双侧肾上腺皮质增生对于患者的预后至关重要。

目前，PA 的诊断分为筛查、确诊及分型三个步骤。国内外指南建议对大部分高血压患者进行 PA 的筛查，包括持续性收缩压>150mmHg 或舒张压>100mmHg、伴有低血钾、伴肾上腺意外瘤的高血压患者等[7-10]。首先通过检测患者醛固酮、肾素并计算醛固酮/肾素比值进行筛查，筛查阳性的患者进一步通过确诊试验（如卡托普利抑制试验、静脉盐水负荷试验等）明确诊断，对于诊断为 PA 的患者，需要进一步分型明确是单侧还是双侧肾上腺病变，以决定是否手术治疗。分型诊断的方法主要包括肾上腺 CT 和肾上腺静脉取血术(AVS)，由于 CT 的准确性只有 70-90%[11-13]，国内外指南均建议，除部分符合典型的 APA 影像学及临床特征（年龄小于 35 岁，伴低血钾病史，且 CT 可见典型的单侧肾上腺腺瘤）的 PA 患者外，其余有手术意愿的 PA 患者均需进一步行 AVS 明确分型，判断醛固酮优势分泌侧，为手术治疗提供依据[7-10]。

AVS 目前主要分为常规 AVS 和促肾上腺皮质激素(ACTH)兴奋的 AVS。肾上腺静脉短而细，且存在解剖变异，此外，肾上腺激素分泌有波动性，这些因素都可能影响 AVS 的成功率，目前国内外报道的 AVS 的成功率为 60-95%[14-16]。由于肾上腺激素存在脉冲式分泌的特点，若取血时激素分泌处于低谷期可能影响结果判断；此外，AVS 除了检测醛固酮还需要检测皮质醇，手术应激对皮质醇有较大影响，这也可能影响结果判断，而 ACTH 刺激可使得肾上腺醛固酮和皮质醇分泌显著增多，有研究显示 ACTH 兴奋可减少激素波动，提高 AVS 成功率[17-18]。课题组前期的回顾性研究也发现，ACTH 兴奋可提高 AVS 成功率，且不影响对肾上腺病变侧的判定[19-20]。但也有研究报道，ACTH 兴奋可能将 PA 患者中单侧肾上腺病变误诊为双侧肾上腺病变，使得患者错失手术机会[21-22]。

综上，ACTH 兴奋的 AVS 可能提高肾上腺静脉插管成功率，但也可能会影响肾上腺醛固酮病变侧的判断。目前，国内外对于常规 AVS 和 ACTH 兴奋的 AVS 对 PA 患者的远期预后的影

响尚未见报道。因此，课题组拟进行一项前瞻性、随机试验，比较基于 ACTH 兴奋的 AVS 和常规的 AVS（即非 ACTH 兴奋的 AVS）判断 PA 患者的醛固酮优势分泌，并确定临床治疗策略，比较 PA 患者的远期预后，从而为临床推荐 AVS 手术方式提供证据。

参考文献

[1] Rossi GP, Bernini G, Caliumi C, et al. A prospective study of the prevalence of primary aldosteronism in 1,125 hypertensive patients[J]. J Am Coll Cardiol 2006; 48(11): 2293-300.

[2] Monticone S, Burrello J, Tizzani D, et al. Prevalence and Clinical Manifestations of Primary Aldosteronism Encountered in Primary Care Practice[J]. Journal of the American College of Cardiology 2017; 69(14): 1811-20.

[3] Xu Zhixin,Yang Jun,Hu Jinbo et al. Primary Aldosteronism in Patients in China With Recently Detected Hypertension.[J] .J Am Coll Cardiol, 2020, 75: 1913-1922.

[4] Mulatero P, Sechi LA, Williams TA, et al. Subtype diagnosis, treatment, complications and outcomes of primary aldosteronism and future direction of research: a position statement and consensus of the Working Group on Endocrine Hypertension of the European Society of Hypertension [J]. Journal of Hypertension, 2020, 38(10): 1929-1936.

[5] Monticone S, D'Ascenzo F, Moretti C, et al. Cardiovascular events and target organ damage in primary aldosteronism compared with essential hypertension: a systematic review and meta-analysis[J]. The Lancet Diabetes & Endocrinology. 2018, 6(1):41-50.

[6] Hundemer Gregory L,Curhan Gary C,Yozamp Nicholas et al. Renal Outcomes in Medically and Surgically Treated Primary Aldosteronism.[J] .Hypertension, 2018, 72: 658-666.

[7] Funder JW, Carey RM, Mantero F, et al. The Management of Primary Aldosteronism: Case Detection, Diagnosis, and Treatment: An Endocrine Society Clinical Practice Guideline[J]. J Clin Endocrinol Metab, 2016, 101(5): 1889-1916.

[8] 原发性醛固酮增多症诊断治疗的专家共识[J]. 中华内分泌代谢杂志, 2016, 32(03): 188-195.

[9] Amar Laurence,Baguet Jean Philippe,Bardet Stéphane et al. SFE/SFHTA/AFCE primary aldosteronism consensus: Introduction and handbook.[J] .Ann Endocrinol (Paris), 2016, 77: 179-86.

[10] Nishikawa T, Omura M, Satoh F, et al. Guidelines for the diagnosis and treatment of primary aldosteronism--the Japan Endocrine Society 2009. Endocrine journal 2011; 58(9): 711-21.

[11] Young William F,Stanson Anthony W,Thompson Geoffrey B et al. Role for adrenal venous sampling in primary aldosteronism.[J] .Surgery, 2004, 136: 1227-35.

[12] Rossi Gian Paolo,Rossitto Giacomo,Amar Laurence et al. Clinical Outcomes of 1625 Patients With Primary Aldosteronism Subtyped With Adrenal Vein Sampling[J] .Hypertension, 2019, 74: 800-808.

[13] Kempers Marlies J E,Lenders Jacques W M,van Outheusden Lieke et al.

Systematic review: diagnostic procedures to differentiate unilateral from bilateral adrenal abnormality in primary aldosteronism.[J] .Ann Intern Med, 2009, 151: 329-37.

[14] Husainy Mohammad Ali,Fang Cheng,Nicolescu Ana et al. Success in adrenal venous sampling between two protocols: experience at a tertiary centre.[J] .J Clin Pathol, 2017, 70: 91-92.

[15] Rossitto Giacomo,Battistel Michele,Barbiero Giulio et al. The subtyping of primary aldosteronism by adrenal vein sampling: sequential blood sampling causes factitious lateralization.[J] .J Hypertens, 2018, 36: 335-343.

[16] Williams TA, Lenders JWM, Mulatero P, et al. Outcomes after adrenalectomy for unilateral primary aldosteronism: an international consensus on outcome measures and analysis of remission rates in an international cohort[J]. Lancet Diabetes Endocrinol. 2017;5(9):689‐699.

[17] Elliott Panda,Holmes Daniel T,Adrenal vein sampling: substantial need for technical improvement at regional referral centres.[J] .Clin Biochem, 2013, 46: 1399-404.

[18] Monticone Silvia,Satoh Fumitoshi,Giacchetti Gilberta et al. Effect of adrenocorticotropic hormone stimulation during adrenal vein sampling in primary aldosteronism.[J] .Hypertension, 2012, 59: 840-6.

[19] 杜志鹏,程庆丰,宋颖,等.探讨促肾上腺皮质激素兴奋在肾上腺静脉取血中的应用价值[J].中华内分泌代谢杂志,2021,37(02):129-134..

[20] Laurent Irakoze,Astère Manirakiza,Zheng Fengfan et al. Adrenal venous sampling with or without adrenocorticotropic hormone stimulation: A meta-analysis.[J] .J Clin Endocrinol Metab, 2018, undefined: undefined.

[21] Seccia, T.M.; Miotto, D.; de Toni, et al. Adrenocorticotropic hormone stimulation during adrenal vein sampling for identifying surgically curable subtypes of primary aldosteronism: Comparison of 3 different protocols[J]. Hypertension 2009, 53, 761‐766.

[22] El Ghorayeb Nada,Mazzuco Tânia L,Bourdeau Isabelle et al. Basal and Post-ACTH Aldosterone and Its Ratios Are Useful During Adrenal Vein Sampling in Primary Aldosteronism.[J] .J Clin Endocrinol Metab, 2016, 101: 1826-35.

二、研究概述

ACTH 兴奋可提高 AVS 插管成功率，但可能影响优势侧判断的准确性，但目前国内外尚无 RCT 研究比较 ACTH 兴奋 AVS 和普通 AVS 对 PA 患者预后的影响。本研究旨在评价基于 ACTH 兴奋 AVS 或普通 AVS，判断 PA 患者有无醛固酮单侧优势分泌，进而确定临床治疗方案，即手术治疗或药物治疗，并通过治疗后随访，比较两组患者的临床预后。终点事件包括完全生化缓解率、高血压缓解情况、生活质量评分及不良事件等。

三、研究设计

（一）研究描述

本研究为随机、对照试验，基于普通 AVS（对照组）或 ACTH 兴奋 AVS，判断 PA 患者有无醛固酮单侧优势分泌，进而确定临床治疗方案，即手术治疗或药物治疗，并长期随访临床转归；确证基于 ACTH 兴奋 AVS 的 PA 患者组预后是否优于基于普通 AVS 组，从而为临床 AVS 的方法选择提供证据。

（二）流程图

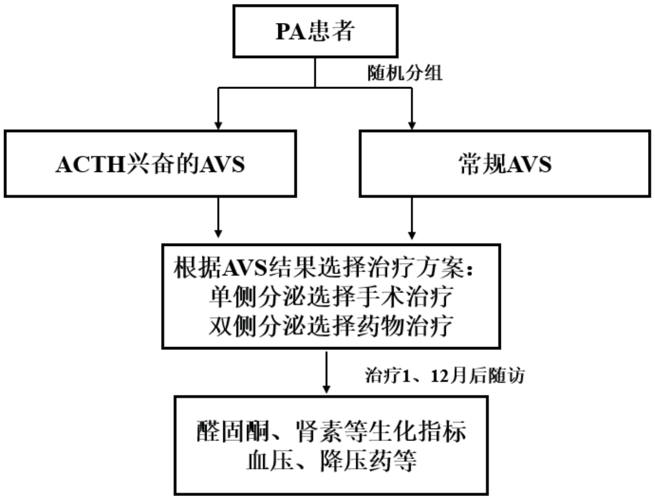

图 1.研究流程图

（三）纳排标准

1、纳入标准

符合下列条件者方可成为本研究的受试者:

- ◆ 了解试验全过程，自愿接受随机化分组、干预及随访；
- ◆ 自愿参加该研究并签署知情同意书，愿意按要求完成所有随访；
- ◆ 年龄 18-70 岁，男女不限，有法定行为能力；
- ◆ 明确诊断为 PA；
- ◆ 在确定醛固酮优势侧分泌情况下，有明确意愿同意接受肾上腺单侧切除术。

2、排除标准

有以下情况之一不能纳入本研究：

- ◆ 孕妇或哺乳期妇女
- ◆ 有未控制的恶性肿瘤患者
- ◆ 对 ACTH 或造影剂过敏；
- ◆ 拒绝接受 AVS 或单侧肾上腺切除手术
- ◆ PA 合并库欣综合征（包括亚临床库欣）；
- ◆ 影像学不除外嗜铬细胞瘤或肾上腺皮质癌；
- ◆ 怀疑家族性醛固酮增多症；

◆ 不适合AVS或单侧肾上腺切除手术，比如合并严重肾功能不全(eGFR<30ml/min/m^2)、严重心功能不全（NYHA 3-4级)、严重贫血(Hb<60g/L)、3月内发生过脑卒中或急性冠脉综合征，严重的腹水及肝硬化等。

◆ 酗酒、吸毒病人，以及有精神障碍不能合作者

（四）PA的诊断

PA筛查均在标准状态下完成，若ARR阳性（ARR>20为阳性，对于有低钾或肾上腺占位的患者，ARR>10为阳性）进一步行确诊试验。

（五）随机对照试验

1、患者来源

有手术意愿的原发性醛固酮增多症患者。

2、受试者分组及随机的实施：

利用SPSS软件中Rv.Uniform函数产生的随机数字表并随机分为ACTH组及非ACTH组，以此确定患者的随机号和组别。

3、干预措施

（1）普通AVS组：术前微量泵入生理盐水（20ml/h)，静滴30min后，分别采集右侧和左侧肾上腺静脉血以及相应的外周静脉血。AVS插管后分别采集右侧和左侧肾上腺静脉血以及相应的外周静脉血，肾上腺皮质醇/外周静脉皮质醇(即选择指数，selectivity index，SI）≥2判定为插管成功。

（2）ACTH兴奋AVS组：术前微量泵入ACTH（250μg即25IU/支，100ml 5-10%葡萄糖配制，50μg/h即20ml/h)，ACTH静滴30min后，分别采集右侧和左侧肾上腺静脉血以及相应的外周静脉血。SI≥3认为插管成功。

在研究过程中，患者和AVS操作医生被实施盲法。患者不知道自己的分组，直到研究结束。为了确保 AVS 操作者不会因分组而影响操作，AVS 操作的医生整个研究过程不知道患者分组。考虑到AVS的结果会泄露患者的分组，AVS结果解读的医生没有实施盲法，但医生需按照实现规定的标准进行判断，标准如下：

对于插管成功者，两侧经肾上腺静脉皮质醇校正过的醛固酮比值(高侧肾上腺静脉血醛固酮/肾上腺皮质醇与低侧肾上腺静脉血醛固酮/肾上腺皮质醇的比值，即优势侧指数，(lateralization index，LI)≥4，建议行单侧肾上腺切除术。LI 2-4的患者如果对侧抑制指数<1或CT提示同侧典型腺瘤，建议手术治疗；LI小于2或LI 2-4但不满足手术条件，则建议药物治疗。

若插管不成功或两侧经肾上腺静脉皮质醇校正过的醛固酮比值均低于外周，若满足以下任何一条建议手术：1)达到bypassAVS条件，即CT单侧>1cm，对侧未见明显结节，无增粗（最宽处小于1cm)，PAC>200pg/ml，PRC<5，K<3.5；2) CT单侧>1cm，对侧未见明显结节，无增粗（最宽处小于1cm)，对侧指数<0.5；若均不满足，则建议药物治疗。

4、资料收集及随访

基线和治疗后第1月及12月收集患者血标本，送检血常规、肾功、电解质、PAC和PRC，必要时重复确诊试验，记录血压及用药情况，测量身高、体重（表1）

表 1. 纳入、干预和评估时间表(ADOPA 研究)

	研究期间			
	纳入	随机分配	随访	
	随访 0	随访 1	1 月(随访 2)	12 月(随访 3)
纳入:				
资格筛选	√			
知情同意	√			
干预措施:				
非促肾上腺皮质激素刺激的肾上腺静脉取血		√		
促肾上腺皮质激素刺激的肾上腺静脉取血		√		
评估:				
体格检查	√		√	√
诊室血压测量	√		√	√
家庭血压监测	√		√	√
药物使用情况	√		√	√
既往史	√			
血常规	√		√	√
肾功能	√		√	√
电解质	√		√	√
立位醛固酮/肾素	√		√	√
确诊试验	√		X	X
心电图	√			
肾上腺 CT	√			
不良事件	√		√	√

X:如果立位醛固酮/肾素阳性，则进行确诊实验；PRC:血浆肾素浓度；PAC:血浆醛固酮浓度；AE：不良事件。

4、终点指标

（1）主要终点

- ◆ 随访 12 个月时，完全生化缓解的比例。

（2）次要终点

- ◆ 随访 12 个月时，临床缓解的比例。
- ◆ 随访 12 个月时，降压药物 DDD 值 (Daily Defined Doses, DDD)。
- ◆ 随访 12 个月时，血压达标率
- ◆ 两组 AVS 插管成功率。
- ◆ 两组间不良事件比较，包括肾上腺静脉出血及相关的肾上腺皮质功能减退、高血压亚急症、过敏性休克、静脉血栓、肺栓塞等。

Original protocol in English

Adrenal venous sampling with or without adrenocorticotropic hormone stimulation in primary aldosteronism: an outcome-based randomized, double-blinded trial (ADOPA study)

I. Research background

Primary aldosteronism (PA), the most common form of secondary hypertension[1-3], is a disease characterized by autonomous aldosterone secretion from adrenocortical lesions and feedback inhibition of the renin-angiotensin system. Aldosterone-producing adenoma (APA) and idiopathic hyperaldosteronism (IHA) are the main types[4]. Unilateral primary aldosteronism (such as APA) can be cured by surgery, while bilateral adrenal hyperplasia cannot obtain sufficient clinical and biochemical benefits through surgery, so medical treatment (such as spironolactone) is recommended. Based on previous studies, patients with surgically treated PA have better long-term outcomes (such as renal function, risk of cardiovascular events, etc.) compared to patients with medically treated PA[5-6]. Overall, we strongly command the early diagnosis and accurate subtyping of patients with PA who are candidates for curative surgical adrenalectomy.

At present, the diagnosis of PA is divided into three steps: screening, diagnosis and subtyping. Guidelines have suggested PA screening for most hypertensive patients, including patients with persistent systolic blood pressure >150mmHg or diastolic blood pressure >100mmHg, hypertension with hypokalemia and adrenal incidentaloma [7-10]. For screening, plasma renin concentration (PRC) and plasma aldosterone concentration (PAC) were measured in the morning. Patients who tested positive should proceed to the confirmatory test(such as captopril inhibition test, saline infusion test, etc.) . All patients with a confirmed diagnosis of PA need further classification to decide whether surgery. The diagnostic methods for classification mainly include adrenal CT and adrenal venous blood collection (AVS). Since the accuracy of CT is only 70-90%[11-13], Patients with PA who are willing to surgery need to undergo adrenal venous sampling (AVS) to determine the predominant secretion side of aldosterone, providing a basis for surgical treatment. except that patients with typical APA (younger than 35 and with spontaneous hypokalemia, marked aldosterone excess, a cortical adenoma on adrenal CT scan) can bypass AVS and proceed to surgery directly[7-10].

At present, there are two protocols of AVS, with or without cosyntropin(ACTH) stimulation.

The adrenal vein is short and thin with anatomical variation. In addition, the secretion of adrenal hormone pulsates. These factors may affect the success rate of AVS, and the success rate of AVS reported is 60-95%[14-16].The pulsatile pattern of secretion of adrenocortical hormones can generate time-related variability in hormone concentrations in the adrenal vein blood, consequently leading to failure of catheterization. In addition, AVS also needs to detect cortisol, and stress has a great impact on cortisol, which may also affect the interpretation of results. ACTH stimulation can significantly increase the secretion of adrenal aldosterone and cortisol, and studies have shown that ACTH stimulation can reduce hormone pulsatile and improve the success rate of AVS [17-18].The previous retrospective study of our group also found that ACTH-stimulated AVS can improve the success rate of AVS without affecting the determination of the adrenal lesion side [19-20]. However,

some studies have reported that ACTH-stimulated AVS may make the wrong interpretation of AVS results, which makes the patient miss the operation opportunity. [21-22].

In short, AVS with ACTH stimulation can facilitate successful catheterization, but it may affect the assessment of lateralization. However, the long-term outcomes of AVS with or without ACTH stimulation in patients with PA has not been evaluated in a randomized controlled study (RCT). Therefore, we set up to a prospective, randomized, double blind, single-center study to evaluate whether the treatment decision (surgical or medical treatment) based on different procedures of AVS (with or without ACTH stimulation) will resulted different outcomes in patients with PA. The results of this study may provide insights into the optimal choice of AVS procedure.

Reference

[1] Rossi GP, Bernini G, Caliumi C, et al. A prospective study of the prevalence of primary aldosteronism in 1,125 hypertensive patients[J]. J Am Coll Cardiol 2006; 48(11): 2293-300.

[2] Monticone S, Burrello J, Tizzani D, et al. Prevalence and Clinical Manifestations of Primary Aldosteronism Encountered in Primary Care Practice[J]. Journal of the American College of Cardiology 2017; 69(14): 1811-20.

[3] Xu Zhixin,Yang Jun,Hu Jinbo et al. Primary Aldosteronism in Patients in China With Recently Detected Hypertension.[J] .J Am Coll Cardiol, 2020, 75: 1913-1922.

[4] Mulatero P, Sechi LA, Williams TA, et al. Subtype diagnosis, treatment, complications and outcomes of primary aldosteronism and future direction of research: a position statement and consensus of the Working Group on Endocrine Hypertension of the European Society of Hypertension [J]. Journal of Hypertension, 2020, 38(10): 1929-1936.

[5] Monticone S, D'Ascenzo F, Moretti C, et al. Cardiovascular events and target organ damage in primary aldosteronism compared with essential hypertension: a systematic review and meta-analysis[J]. The Lancet Diabetes & Endocrinology. 2018, 6(1):41-50.

[6] Hundemer Gregory L,Curhan Gary C,Yozamp Nicholas et al. Renal Outcomes in Medically and Surgically Treated Primary Aldosteronism.[J] .Hypertension, 2018, 72: 658-666.

[7] Funder JW, Carey RM, Mantero F, et al. The Management of Primary Aldosteronism: Case Detection, Diagnosis, and Treatment: An Endocrine Society Clinical Practice Guideline[J]. J Clin Endocrinol Metab, 2016, 101(5): 1889-1916.

[8] Consensus on diagnosis and treatment of primary aldosteronism [J]. Zhong Hua Nei Fen Mi Dai Xie Za Zhi,2016, 32(03): 188-195.

[9] Amar Laurence,Baguet Jean Philippe,Bardet Stéphane et al. SFE/SFHTA/AFCE primary aldosteronism consensus: Introduction and handbook.[J] .Ann Endocrinol (Paris), 2016, 77: 179-86.

[10] Nishikawa T, Omura M, Satoh F, et al. Guidelines for the diagnosis and treatment of primary aldosteronism--the Japan Endocrine Society 2009. Endocrine journal 2011; 58(9): 711-21.

[11] Young William F,Stanson Anthony W,Thompson Geoffrey B et al. Role for adrenal venous sampling in primary aldosteronism.[J] .Surgery, 2004, 136: 1227-35.

[12] Rossi Gian Paolo,Rossitto Giacomo,Amar Laurence et al. Clinical Outcomes of 1625 Patients With Primary Aldosteronism Subtyped With Adrenal Vein Sampling[J] .Hypertension, 2019, 74: 800-808.

[13] Kempers Marlies J E,Lenders Jacques W M,van Outheusden Lieke et al. Systematic review: diagnostic procedures to differentiate unilateral from bilateral adrenal abnormality in primary

aldosteronism.[J] .Ann Intern Med, 2009, 151: 329-37.
[14] Husainy Mohammad Ali,Fang Cheng,Nicolescu Ana et al. Success in adrenal venous sampling between two protocols: experience at a tertiary centre.[J] .J Clin Pathol, 2017, 70: 91-92.
[15] Rossitto Giacomo,Battistel Michele,Barbiero Giulio et al. The subtyping of primary aldosteronism by adrenal vein sampling: sequential blood sampling causes factitious lateralization.[J] .J Hypertens, 2018, 36: 335-343.
[16] Williams TA, Lenders JWM, Mulatero P, et al. Outcomes after adrenalectomy for unilateral primary aldosteronism: an international consensus on outcome measures and analysis of remission rates in an international cohort[J]. Lancet Diabetes Endocrinol. 2017;5(9):689-699.
[17] Elliott Panda,Holmes Daniel T,Adrenal vein sampling: substantial need for technical improvement at regional referral centres.[J] .Clin Biochem, 2013, 46: 1399-404.
[18] Monticone Silvia,Satoh Fumitoshi,Giacchetti Gilberta et al. Effect of adrenocorticotropic hormone stimulation during adrenal vein sampling in primary aldosteronism.[J] .Hypertension, 2012, 59: 840-6.
[19] Du Zhipeng, Chen Qingfeng, Song Ying et al.Effect of adrenocorticotropic hormone stimulation during adrenal vein sampling [J].Zhong Hua Nei Fen Mi Dai Xie Za Zhi,2021,37(02):129-134..
[20] Laurent Irakoze,Astère Manirakiza,Zheng Fengfan et al. Adrenal venous sampling with or without adrenocorticotropic hormone stimulation: A meta-analysis.[J] .J Clin Endocrinol Metab, 2018, undefined: undefined.
[21] Seccia, T.M.; Miotto, D.; de Toni, et al. Adrenocorticotropic hormone stimulation during adrenal vein sampling for identifying surgically curable subtypes of primary aldosteronism: Comparison of 3 different protocols[J]. Hypertension 2009, 53, 761–766.
[22] El Ghorayeb Nada,Mazzuco Tânia L,Bourdeau Isabelle et al. Basal and Post-ACTH Aldosterone and Its Ratios Are Useful During Adrenal Vein Sampling in Primary Aldosteronism.[J] .J Clin Endocrinol Metab, 2016, 101: 1826-35.

II Research overview

Adrenocorticotropic hormone (ACTH) is used during AVS in some centers due to reported benefit of increasing the rates of successful adrenal vein cannulation, but it may affect the accuracy of judging lateralization. The outcomes of treatment following AVS with or without ACTH stimulation have not been evaluated in a prospective study. Therefore, we set up to a randomized clinical trial to evaluate whether the treatment decision (surgical or medical treatment) based on different AVS procedures (with or without ACTH stimulation) will lead to different outcomes in patients with PA. The endpoint included complete biochemical remission rate, hypertension remission, quality of life score and adverse events.

III Research design

(1) Description of the study

This study is randomized, double-blinded (patients and AVS performers), controlled clinical

trial to evaluate whether the treatment decision (surgical or medical treatment) based on different AVS procedures, namely with (controlled group) and without ACTH stimulation, will lead to different outcomes in patients with PA. It is aimed to confirm whether clinical outcomes based on ACTH stimulated AVS is better than that based on non-ACTH stimulated AVS. The results of this study may provide insights into the value of ACTH stimulation in AVS for PA subtyping.

(2) Flow chart

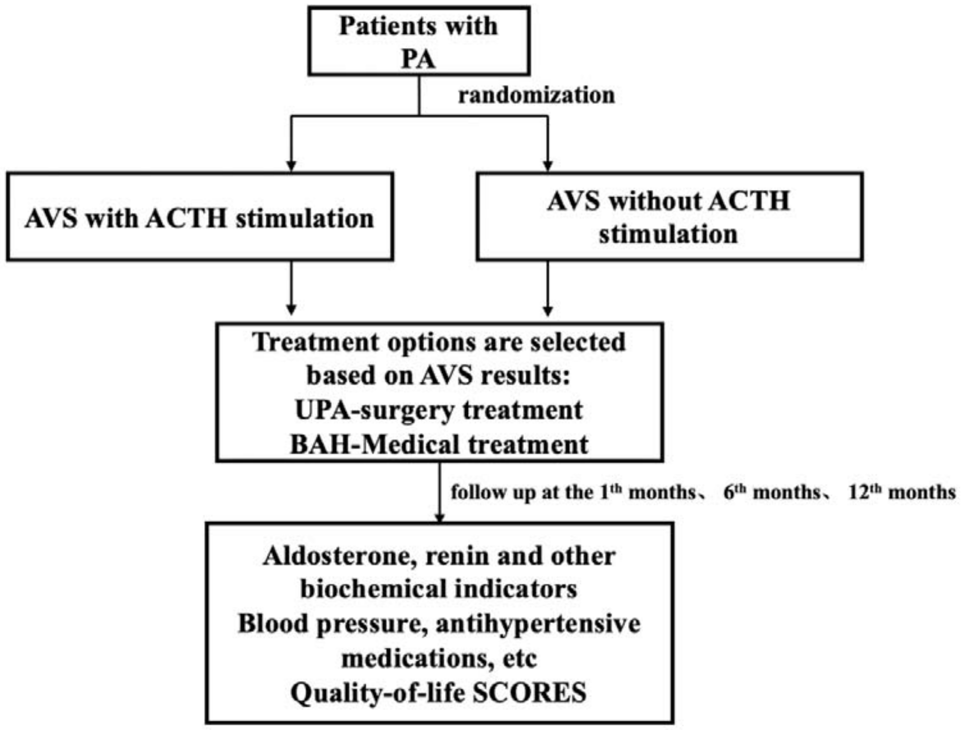

Figure 1. Research flow chart

(3) Eligibility

1 .Inclusion criteria:

Patients who meet the following criterion can be included in this study:

- Understand the whole process of the trial and voluntarily accept randomized grouping, intervention and follow-up
- Voluntarily participate in the study and sign the informed consent form
- Above the age of 18, male or female, with legal capacity
- Hypertension patients with positive PA screening (ARR>20) and at least one positive PA confirmatory test (CCT, SSIT, FST)
- Under the condition of determining lateralization, there is a clear willingness to accept unilateral adrenalectomy

2. Exclusion criteria

Patients with one of the following conditions will be excluded in this study:

- Pregnant or lactating women
- People with a history of malignant tumor
- Not suitable or refusal to undergo AVS or unilateral adrenalectomy, or allergic to ACTH
- PA complicated with Cushing syndrome (including subclinical Cushing)
- Consider glucocorticoid-induced aldosteronism (GRA) or adrenocortical carcinoma
- Cannot stop or change the medicine, and the drug has a related effect with the treatment prescribed in this study, or it will lead to hypertension, such as glucocorticoid.
- Complicated with disease which contraindicated to unilateral adrenalectomy or seriously interfere with subsequent treatment of PA or health-related quality of life assessment, such as severe heart disease (including pacemaker implantation), severe cardiac dysfunction, severe anemia (Hb<60g/L), stroke or acute coronary syndrome within 3 months, severe ascites and liver cirrhosis at baseline
- Alcoholics, drug users, and mentally disabled patients.

3. Rejection criteria

Patient with one of the following conditions will be withdraw from the trial:

- The patient did not choose the treatment according to the research standard or lost follow-up
- Serious CVD, such as myocardial infarction, stroke and severe cardiac insufficiency occurred
- Severe renal insufficiency occurred

(4) Randomized controlled trials

1. Patients

Patients with primary aldosteronism who are willing to undergo surgery.

2. Randomization

Using the random number table generated by the Rv.Uniform function in SPSS software and randomly divided into ACTH-stimulated group(Intervention group) and ACTH-unstimulated group(Control group), in order to determine the random number and group of patients.

3. Intervention methods

(1) AVS without ACTH stimulation: Right and left adrenal venous blood and corresponding peripheral venous blood should be sampled sequentially. Cannulation was considered successful when the selectivity index (SI), namely plasma cortisol concentration (PCC) in adrenal vein/PCC in IVC≥2. The ratio of PAC: PCC on the side with the higher ratio over the contralateral PAC: PCC ratio is defined as the lateralization index (LI). Lateralization of aldosterone excess was defined as LI≥4 suggested unilateral adrenalectomy.

(2) AVS with ACTH stimulation: ACTH (250μg, 25IU/ branch, 100 ml of 5-10% glucose, 50μg/h, 20ml/h) was administered as continuous infusion which was started 30 minutes before sampling and continued throughout the procedure. Right and left adrenal venous blood and corresponding peripheral venous blood should be sampled sequentially. SI≥3 considered cannulation successful; LI≥4 suggests unilateral adrenalectomy.

4. Data collection and follow-up

Blood samples of patients will be collected at baseline and 1, 6 and 12 months after the intervention, and routine blood tests, kidney function, electrolyte, PAC and PRC will be detected. Confirmatory tests should be repeated when necessary. Blood pressure, medication and quality of life scores will be recorded, height and weight will be measured. (Table 1).

Table 1. Follow-up Flow Chart

parameter	baseline	1st (V1)	6th (V2)	12th (V3)
**General information**				
Demographic data	√			
History of disease	√			√
**Clinical Characteristics**				
height	√			√
weight	√			√
waist	√			√
blood pressure	√	√	√	√
Antihypertensive medications	√	√	√	√
**Biochemical test**				
electrolytic	√	√	√	√
kidney function	√	√	√	√
Blood routine test	√	√	√	√
PAC/PRC	√	√	√	√
confirmatory test	√	**?**		**?**
life quality score	√			√

? When necessary

5. Outcomes

(1) Primary outcome

- The proportion of complete biochemical remission at 12th months of follow-up

(2) Secondary outcome

- The proportion of clinical remission at 12 months of follow-up
- Daily defined doses (DDD) of antihypertensive agents, achieved a target blood pressure of 140/90 mmHg or below at 12 months of follow-up
- The rate of successful catheterization

- Life quality score at 12 months of follow-up

Final protocol in English

Adrenal venous sampling with or without adrenocorticotropic hormone stimulation in primary aldosteronism: an outcome-based randomized trial (ADOPA study)

I. Research background

Primary aldosteronism (PA), the most common form of secondary hypertension[1-3], is a disease characterized by autonomous aldosterone secretion from adrenocortical lesions and feedback inhibition of the renin-angiotensin system. Aldosterone-producing adenoma (APA) and idiopathic hyperaldosteronism (IHA) are the main types[4]. Unilateral primary aldosteronism (such as APA) can be cured by surgery, while bilateral adrenal hyperplasia cannot obtain sufficient clinical and biochemical benefits through surgery, so medical treatment (such as spironolactone) is recommended. Based on previous studies, patients with surgically treated PA have better long-term outcomes (such as renal function, risk of cardiovascular events, etc.) compared to patients with medically treated PA[5-6]. Overall, we strongly command the early diagnosis and accurate subtyping of patients with PA who are candidates for curative surgical adrenalectomy.

At present, the diagnosis of PA is divided into three steps: screening, diagnosis and subtyping. Guidelines have suggested PA screening for most hypertensive patients, including patients with persistent systolic blood pressure >150mmHg or diastolic blood pressure >100mmHg, hypertension with hypokalemia and adrenal incidentaloma [7-10]. For screening, plasma renin concentration (PRC) and plasma aldosterone concentration (PAC) were measured in the morning. Patients who tested positive should proceed to the confirmatory test(such as captopril inhibition test, saline infusion test, etc.) . All patients with a confirmed diagnosis of PA need further classification to decide whether surgery. The diagnostic methods for classification mainly include adrenal CT and adrenal venous blood collection (AVS). Since the accuracy of CT is only 70-90%[11-13], Patients with PA who are willing to surgery need to undergo adrenal venous sampling (AVS) to determine the predominant secretion side of aldosterone, providing a basis for surgical treatment. except that patients with typical APA (younger than 35 and with spontaneous hypokalemia, marked aldosterone excess, a cortical adenoma on adrenal CT scan) can bypass AVS and proceed to surgery directly[7-10].

At present, there are two protocols of AVS, with or without cosyntropin(ACTH) stimulation.

The adrenal vein is short and thin with anatomical variation. In addition, the secretion of adrenal hormone pulsates. These factors may affect the success rate of AVS, and the success rate of AVS reported is 60-95%[14-16].The pulsatile pattern of secretion of adrenocortical hormones can generate time-related variability in hormone concentrations in the adrenal vein blood, consequently leading to failure of catheterization. In addition, AVS also needs to detect cortisol, and stress has a great impact on cortisol, which may also affect the interpretation of results. ACTH stimulation can significantly increase the secretion of adrenal aldosterone and cortisol, and studies have shown that ACTH stimulation can reduce hormone pulsatile and improve the success rate of AVS [17-18].The previous retrospective study of our group also found that ACTH-stimulated AVS can improve the success rate of AVS without affecting the determination of the adrenal lesion side [19-20]. However,

some studies have reported that ACTH-stimulated AVS may make the wrong interpretation of AVS results, which makes the patient miss the operation opportunity. [21-22].

In short, AVS with ACTH stimulation can facilitate successful catheterization, but it may affect the assessment of lateralization. However, the long-term outcomes of AVS with or without ACTH stimulation in patients with PA has not been evaluated in a randomized controlled study (RCT). Therefore, we set up to a prospective, randomized, double blind, single-center study to evaluate whether the treatment decision (surgical or medical treatment) based on different procedures of AVS (with or without ACTH stimulation) will resulted different outcomes in patients with PA. The results of this study may provide insights into the optimal choice of AVS procedure.

Reference

[1] Rossi GP, Bernini G, Caliumi C, et al. A prospective study of the prevalence of primary aldosteronism in 1,125 hypertensive patients[J]. J Am Coll Cardiol 2006; 48(11): 2293-300.

[2] Monticone S, Burrello J, Tizzani D, et al. Prevalence and Clinical Manifestations of Primary Aldosteronism Encountered in Primary Care Practice[J]. Journal of the American College of Cardiology 2017; 69(14): 1811-20.

[3] Xu Zhixin,Yang Jun,Hu Jinbo et al. Primary Aldosteronism in Patients in China With Recently Detected Hypertension.[J] .J Am Coll Cardiol, 2020, 75: 1913-1922.

[4] Mulatero P, Sechi LA, Williams TA, et al. Subtype diagnosis, treatment, complications and outcomes of primary aldosteronism and future direction of research: a position statement and consensus of the Working Group on Endocrine Hypertension of the European Society of Hypertension [J]. Journal of Hypertension, 2020, 38(10): 1929-1936.

[5] Monticone S, D'Ascenzo F, Moretti C, et al. Cardiovascular events and target organ damage in primary aldosteronism compared with essential hypertension: a systematic review and meta-analysis[J]. The Lancet Diabetes & Endocrinology. 2018, 6(1):41-50.

[6] Hundemer Gregory L,Curhan Gary C,Yozamp Nicholas et al. Renal Outcomes in Medically and Surgically Treated Primary Aldosteronism.[J] .Hypertension, 2018, 72: 658-666.

[7] Funder JW, Carey RM, Mantero F, et al. The Management of Primary Aldosteronism: Case Detection, Diagnosis, and Treatment: An Endocrine Society Clinical Practice Guideline[J]. J Clin Endocrinol Metab, 2016, 101(5): 1889-1916.

[8] Consensus on diagnosis and treatment of primary aldosteronism [J]. Zhong Hua Nei Fen Mi Dai Xie Za Zhi,2016, 32(03): 188-195.

[9] Amar Laurence,Baguet Jean Philippe,Bardet Stéphane et al. SFE/SFHTA/AFCE primary aldosteronism consensus: Introduction and handbook.[J] .Ann Endocrinol (Paris), 2016, 77: 179-86.

[10] Nishikawa T, Omura M, Satoh F, et al. Guidelines for the diagnosis and treatment of primary aldosteronism--the Japan Endocrine Society 2009. Endocrine journal 2011; 58(9): 711-21.

[11] Young William F,Stanson Anthony W,Thompson Geoffrey B et al. Role for adrenal venous sampling in primary aldosteronism.[J] .Surgery, 2004, 136: 1227-35.

[12] Rossi Gian Paolo,Rossitto Giacomo,Amar Laurence et al. Clinical Outcomes of 1625 Patients With Primary Aldosteronism Subtyped With Adrenal Vein Sampling[J] .Hypertension, 2019, 74: 800-808.

[13] Kempers Marlies J E,Lenders Jacques W M,van Outheusden Lieke et al. Systematic review: diagnostic procedures to differentiate unilateral from bilateral adrenal abnormality in primary

aldosteronism.[J] .Ann Intern Med, 2009, 151: 329-37.
[14] Husainy Mohammad Ali,Fang Cheng,Nicolescu Ana et al. Success in adrenal venous sampling between two protocols: experience at a tertiary centre.[J] .J Clin Pathol, 2017, 70: 91-92.
[15] Rossitto Giacomo,Battistel Michele,Barbiero Giulio et al. The subtyping of primary aldosteronism by adrenal vein sampling: sequential blood sampling causes factitious lateralization.[J] .J Hypertens, 2018, 36: 335-343.
[16] Williams TA, Lenders JWM, Mulatero P, et al. Outcomes after adrenalectomy for unilateral primary aldosteronism: an international consensus on outcome measures and analysis of remission rates in an international cohort[J]. Lancet Diabetes Endocrinol. 2017;5(9):689-699.
[17] Elliott Panda,Holmes Daniel T,Adrenal vein sampling: substantial need for technical improvement at regional referral centres.[J] .Clin Biochem, 2013, 46: 1399-404.
[18] Monticone Silvia,Satoh Fumitoshi,Giacchetti Gilberta et al. Effect of adrenocorticotropic hormone stimulation during adrenal vein sampling in primary aldosteronism.[J] .Hypertension, 2012, 59: 840-6.
[19] Du Zhipeng, Chen Qingfeng, Song Ying et al.Effect of adrenocorticotropic hormone stimulation during adrenal vein sampling [J].Zhong Hua Nei Fen Mi Dai Xie Za Zhi,2021,37(02):129-134..
[20] Laurent Irakoze,Astère Manirakiza,Zheng Fengfan et al. Adrenal venous sampling with or without adrenocorticotropic hormone stimulation: A meta-analysis.[J] .J Clin Endocrinol Metab, 2018, undefined: undefined.
[21] Seccia, T.M.; Miotto, D.; de Toni, et al. Adrenocorticotropic hormone stimulation during adrenal vein sampling for identifying surgically curable subtypes of primary aldosteronism: Comparison of 3 different protocols[J]. Hypertension 2009, 53, 761–766.
[22] El Ghorayeb Nada,Mazzuco Tânia L,Bourdeau Isabelle et al. Basal and Post-ACTH Aldosterone and Its Ratios Are Useful During Adrenal Vein Sampling in Primary Aldosteronism.[J] .J Clin Endocrinol Metab, 2016, 101: 1826-35.

II Research overview

Adrenocorticotropic hormone (ACTH) is used during AVS in some centers due to reported benefit of increasing the rates of successful adrenal vein cannulation, but it may affect the accuracy of judging lateralization. The outcomes of treatment following AVS with or without ACTH stimulation have not been evaluated in a prospective study. Therefore, we set up to a randomized clinical trial to evaluate whether the treatment decision (surgical or medical treatment) based on different AVS procedures (with or without ACTH stimulation) will lead to different outcomes in patients with PA. The endpoint included complete biochemical remission rate, hypertension remission, quality of life score and adverse events.

III Research design

(1) Description of the study

This study is randomized, controlled clinical trial to evaluate whether the treatment decision

(surgical or medical treatment) based on different AVS procedures, namely with (controlled group) and without ACTH stimulation, will lead to different outcomes in patients with PA. It is aimed to confirm whether clinical outcomes based on ACTH stimulated AVS is better than that based on non-ACTH stimulated AVS. The results of this study may provide insights into the value of ACTH stimulation in AVS for PA subtyping.

(2) Flow chart

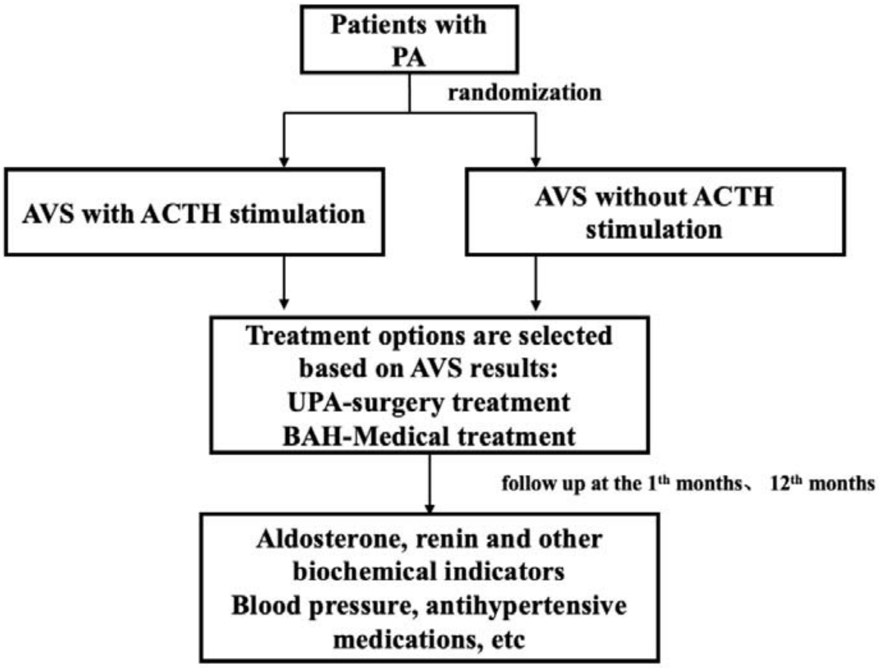

Figure 1. Research flow chart

(3) Eligibility

1.Inclusion criteria:

Patients who meet the following criterion can be included in this study:

- ◆ Understand the whole process of the trial and voluntarily accept randomized grouping, intervention and follow-up
- ◆ Voluntarily participate in the study and sign the informed consent form
- ◆ Aged between 18-70, male or female, with legal capacity
- ◆ Definite diagnosis of PA
- ◆ Under the condition of determining lateralization, there is a clear willingness to accept unilateral adrenalectomy

2. Exclusion criteria

Patients with one of the following conditions will be excluded in this study:

- Pregnant or lactating women
- Patients with a history of uncontrolled malignant tumor
- Allergic to ACTH or contrast media
- Refusal by the patient to undergo AVS or adrenalectomy
- Complicated with Cushing's syndrome (including subclinical Cushing)
- With imaging characteristics suggestive of pheochromocytoma or adrenal cortical carcinoma
- Diagnosed with familial hyperaldosteronism
- Patients unsuitable for surgery, such as those with heart failure (New York Heart Association (NYHA) class III or IV), severe anemia (Hemoglobin<60g/L), stroke or acute coronary syndrome within 3 months, severe ascites and cirrhosis, estimated glomerulus filtration rate<30ml/min/m2
- With alcohol or drug abuse and active mental health disorders.

(4) Diagnosis of PA

All hypertensive patients will undergo PA screening by plasma aldosterone/renin ratio (ARR). PA screening will be completed in standard condition: before screening, diuretic therapy, including mineralocorticoid receptor antagonists (MRA), will be withdrawn for at least 4 weeks, and angiotensin-converting enzyme inhibitors, angiotensin-II receptor blockers and β-blockers will be stopped for at least two weeks. Non-dihydropyridine calcium channel blockers and/or α-adrenergic blockers are allowed for uncontrolled hypertension. Samples for plasma renin concentration (PRC) and plasma aldosterone concentration (PAC) will be collected in the morning after participants had been out of bed for at least 2 hours and after they had been seated for 15 minutes. The screening test will be considered positive when the ARR is ≥ 2.0 $ng.dl^{-1}/IU.l^{-1}$. Patients who test positive will proceed to confirmatory testing. For patients who test negative with the ARR, if PA is strongly suspected based on young age, hypokalemia, or resistant hypertension, they will also proceed to the confirmatory test. PA will be confirmed if at least one of the following criteria is met: 1) PAC ≥11 ng/dl two hours after administration of 50 mg captopril; 2) PAC ≥8.0 ng/dl after the infusion of 2L normal saline; 3) if confirmatory tests in grey zone (i.e, PAC 8-11 ng/dl two hours after administration of 50 mg captopril or PAC 6-8 ng/dl after the infusion of 2L normal saline), we will further perform fludrocortisone suppression test and diagnose as PA if PAC on the fourth day of fludrocortisone administration exceeds 6.0 ng/dl1.

Patients with confirmed PA then will undergo thin-slice (1mm thick) adrenal CT. The CT findings are classified into unilateral lesion, bilaterally normal, and bilateral lesions. Adrenal lesion includes nodule (defined as round or oval, with smooth margins, well defined, ≥4mm in diameter) and hyperplasia (if adrenal gland thickness measured ≥10 mm in diameter). If no nodule or hyperplasia is found, the CT result is considered as normal.

(5) Randomized controlled trials

1. Patients

Patients with primary aldosteronism who are willing to undergo surgery.

2. Randomization

Using the random number table generated by the Rv.Uniform function in SPSS software and randomly divided into ACTH-stimulated group(Intervention group) and ACTH-unstimulated group(Control group), in order to determine the random number and group of patients.

3. Intervention methods

(1) AVS without ACTH stimulation: Normal saline will be administered as continuous infusion which is started 30 minutes before sampling and continues throughout the procedure at 20ml/hr. Blood samples will be collected sequentially from right and left adrenal veins, and blood in the inferior vena cava will be collected immediately after the collection of each side of the adrenal vein blood. Cannulation will be considered successful when the selectivity index (SI), namely plasma cortisol concentration (PCC) in adrenal vein/PCC in IVC≥2.

(2) AVS with ACTH stimulation: ACTH (250μg, 25IU/ branch, 100 ml of 5-10% glucose, 50μg/h, 20ml/h) will be administered as continuous infusion which is started 30 minutes before sampling and continues throughout the procedure. Right and left adrenal venous blood and corresponding peripheral venous blood should be sampled sequentially. SI≥3 will be considered cannulation successful.

In this study, the patients and AVS performers will be blinded. Patients will be blinded until the end of the study. To ensure that the AVS operator will not influence the operation by knowing grouping, the AVS operator will be blinded throughout the study. Due to AVS results may reveal the grouping, therefore, the investigators analyzing AVS results will not be blinded. The physicians are required to judgment according to the specified criteria for implementation, as follows:

For patients with successful cannulation, the ratio of PAC: PCC on the side with the higher ratio over the contralateral PAC: PCC ratio is defined as the lateralization index (LI). Lateralization of aldosterone excess is defined as LI≥4 irrespective of ACTH use suggested unilateral adrenalectomy. Patients with LI between 2 and 4 together with contralateral suppression (PAC/PCC of non-dominant side < PAC/PCC of IVC) or CT showing a typical adenoma on the dominant side will be also considered to have lateralized disease suggested unilateral adrenalectomy. Patients with LI < 2 or LI 2-4 without meeting the above criteria will be diagnosed as BPA suggested treated with medications.

In case of technical AVS failure or bilateral PAC/PCC in adrenal venous blood lower than peripheral blood, adrenalectomy will be recommended if the patient meets one of the following criteria: 1) unilateral nodule on CT (≥ 1 cm), no observable nodules or hyperplasia on contralateral adrenal, and PAC ≥ 20 ng/dl, PRC < 5 mIU/l, K ≤ 3.5mmol/l; 2) unilateral nodule on CT (≥ 1 cm), no observable nodules or hyperplasia on contralateral adrenal, and the contralateral index≤0.5 in AVS.

4. Data collection and follow-up

Blood samples of patients will be collected at baseline and 1, 6 and 12 months after the intervention, and routine blood tests, kidney function, electrolyte, PAC and PRC will be detected. Confirmatory tests should be repeated when necessary. Blood pressure, medication and quality of

life scores will be recorded, height and weight will be measured. (Table 1).

Table1. The schedule of enrolment, interventions, and assessments (ADOPA study)

	Study period			
	Enrolment	Allocation	Follow-up	
	Visit 0	Visit 1	1st Month (Visit 2)	12th Month (Visit 3)
Enrolment:				
Eligibility screen	√			
Informed consent	√			
Interventions:				
non-ACTH stimulated AVS		√		
ACTH stimulated AVS		√		
Assessments:				
Physical examinations	√		√	√
Office Blood Pressure measurement	√		√	√
Home Blood Pressure Monitoring	√		√	√
Drugs use record	√		√	√
History of diseases	√			
Blood routine	√		√	√
Renal function test	√		√	√
Electrolytes	√		√	√
Upright PAC/PRC	√		√	√
Confirmatory test	√		X	X
EGC	√			
Adrenal CT	√			
Recording of AEs	√		√	√

X: If Upright PAC/PRC was positive, captopril challenge test or seated saline infusion test were performed. PRC: plasma renin concentration; PAC: plasma aldosterone concentration; EGC: electrocardiogram; AE: adverse Event.

5. Outcomes

(1) Primary outcome

- The proportion of complete biochemical remission at 12 th months of follow-up

(2) Secondary outcome

- The proportion of clinical remission at 12 months of follow-up
- Daily defined doses (DDD) of antihypertensive agents at 12 months of follow-up
- Proportion of patients reaching target blood pressure at 12 months of follow-up

- The rate of successful catheterization
- Adverse events, including adrenal venous hemorrhage and related adrenal insufficiency,

Statistical analysis plan in Chinese

统计分析计划

一般方法和注意事项

我们将总结每个干预组的基线特征，以及受试者的处置情况和终止原因。

研究终点

研究的主要终点是在 12 个月的随访时，整个队列中手术治疗后生化完全缓解的患者比例。

次要结局包括:1)在随访 12 个月时，手术治疗患者在整个队列中达到完全临床缓解的比例;2)随访 12 个月时，各组降压药(包括 MRA)的日限定剂量(DDD)、血压、达到目标血压的患者比例;3)研究过程中的不良事件;4)双侧 AVS 成功率。

根据原发性醛固酮增多症手术结果(PASO)标准确定生化和临床缓解。DDD 是假定的用于成人的药物每天的平均维持剂量。例如，5 毫克氨氯地平的 DDD 为 1,10 毫克福辛普利也是如此。如果两种药物同时服用，则 DDD 为 2。

终点分析

在意向诊断的基础上进行分析:患者将在随机分配到的诊断组中进行分析，AVS 失败的患者将留在随机分配到的组中，即使他们的治疗方法不是由 AVS 决定的。

亚组分析包括：1)排除违反治疗决策方案的患者:诊断为 UPA 但经药物治疗或诊断为 BPA 但经肾上腺切除术治疗;2)排除 AVS 失败患者;3) ACTH 刺激组 AVS 选择性指数由 3 提高到 5，非 ACTH 刺激组 AVS 选择性指数由 2 提高到 3;4)将 LI 从 4 降低到 2。

其它分析

实验室结果:通过使用平均值、标准差、中位数、最小值和最大值对实验室结果进行描述。还可以提供汇总数据的图形进行表示。为了评估两组之间差异的显著性，我们将对分类数据使用 χ 2 检验或 Fisher 精确检验，对具有和不具有正态分布的连续数据分别使用非配对 t 检验和 Mann-Whitney U 检验。P 值小于 0.05 为显著性。我们将使用 IBM SPSS statistics 20 进行统计分析。

样本量计算

根据以下假设，该试验设计有 80%的能力检测两组之间 19%的完全生化缓解比例的差异:与不刺激 ACTH 的 AVS 相比，刺激 ACTH 的 AVS 使 PA 患者插管失败的次数减少了 28%。根据我们之前的研究，我们中心接受 AVS 的患者中有 70%是 UPA, 93%的 UPA 在肾上腺切除术后取得了完全的生化缓解。我们假设未刺激 ACTH 的 AVS 插管成功率为 70%，未刺激 ACTH 的 AVS 完全生化缓解率为 0.7*0.7*0.93=0.45，刺激 ACTH 的 AVS 完全生化缓解率为 0.7*(0.7+0.28)*0.93= 0.64。双侧显著性水平为 0.05，总样本量为 208 例，每组 104 例，则有 80%的能力检测两组之间 19%的完全生化缓解比例的差异。估计约 10%的退出率，我们的目标是每组 115 例患者的样本量。

Statistical analysis plan in English

Statistical analysis plan

General Approach/Considerations

Baseline characteristics will be summarized for each treatment group. Subject disposition and reason for discontinuation will be summarized for each group.

Outcomes

The primary endpoint was the proportion of surgically treated patients with complete biochemical remission in the overall cohort after 12 months of follow-up.

The secondary outcomes including: 1) the proportion of surgically treated patients who achieved complete clinical remission in the overall cohort after 12 months of follow-up; 2) daily defined doses (DDD) of antihypertensive agents (including MRA), blood pressure, proportion of patients reaching target blood pressure in each group irrespective of their treatment after 12 months of follow-up; 3) adverse events during the study; 4) the rate of bilaterally successful AVS.

Biochemical and clinical remission were defined based on the Primary Aldosteronism Surgery Outcome (PASO) criteria. DDD is the assumed average maintenance dose per day for a drug used for its main indication in adults. For instance, 5 mg of amlodipine has a DDD of one, as does 10 mg of fosinopril. If both drugs are taken together, the DDD is two.

Analysis of endpoints

Analysis will be conducted based on an intention-to-diagnose basis: patients will be analyzed in the diagnostic group to which they have been randomly assigned and the patients with failed AVS remained in the group to which they have been randomly assigned, even though their treatment is not determined by AVS.

Subgroup analysis were conducted 1) when the patient who violate the treatment decision protocol were excluded: if the patient was diagnosed as UPA but treated with medication or if diagnosed as BPA but treated with adrenalectomy; 2) when the patients with failed AVS were excluded; 3) increasing the AVS selectivity index from 3 to 5 in the ACTH stimulated group and from 2 to 3 in the non-ACTH stimulated group; 4) decreasing LI from 4 to 2.

Additional Analyses

Laboratory Values: Laboratory variables will be summarized by assessment visit using mean, standard deviation, median, minimum, and maximum. Graphical representations of aggregate data may also be presented. To assess significance of differences between two groups, we will use χ^2 test or Fisher's exact test for categorical data and unpaired t test and Mann-Whitney U test for continuous data with and without a normal distribution, respectively. P value less than 0·05 will be considered significant. We will use IBM SPSS statistics 20 for statistical analysis.

Sample size calculation

The trial was designed to have a power of 80% to detect a difference between the two groups in the proportion of complete biochemical remission of 19%, based on the following assumptions: compared with AVS without ACTH stimulation, AVS with ACTH stimulation reduced the number of unsuccessful cannulations in patients with PA by 28%6.Based on our previous research3, 70% of the patients in our center underwent AVS was UPA, and 93% UPA achieved complete biochemical success post-adrenalectomy. We assumed that, successful cannulation rate was 70% in AVS without ACTH stimulation, and the complete biochemical success rate would be 0.7*0.7*0.93=0.45 in AVS without ACTH stimulation and 0.7*(0.7+0.28)*0.93= 0.64 in AVS with ACTH stimulation. Calculation of the sample size, with a two-sided significance level of 0.05, indicates a required total sample size of 208 patients,

104 in each group, having a power of 80% to detect a difference between the two groups in the proportion of complete biochemical remission of 19%. To account for a ~10 % drop out rate we aimed at a sample size of 115 patients.

Patient informed consent in Chinese

ACTH 兴奋和非 ACTH 兴奋的肾上腺静脉取血对原发性醛固酮增多症患者的影响：一项基于临床结局的随机、双盲研究

患者知情同意书

研究背景介绍：

尊敬的患者，您被邀请参加重庆医科大学附属第一医院内分泌科开展的“ACTH 兴奋和非 ACTH 兴奋的肾上腺静脉取血（AVS）对原发性醛固酮增多症（PA）患者的影响：一项基于临床结局的随机、双盲研究”。

目前，PA 的诊断分为筛查、确诊及分型三个步骤。国内外指南均建议有手术意愿的 PA 患者均需进一步行 AVS 判断醛固酮优势分泌侧，为手术治疗提供依据。AVS 目前主要分为常规 AVS（即非 ACTH 兴奋的 AVS）和 ACTH 兴奋的 AVS。ACTH 兴奋的 AVS 可能提高肾上腺静脉插管成功率，但也可能会影响肾上腺醛固酮病变侧的判断。

该项目旨在比较基于 ACTH 兴奋的 AVS 和常规的 AVS 判断 PA 患者的醛固酮优势分泌，并确定临床治疗策略，比较 PA 患者的远期预后。本知情同意书提供您一些信息，以帮助您决定是否参加此项研究。您参加本研究是自愿的，本项目已通过本医院伦理审查委员会审查，如果您同意加入此项目，请查看下列说明：

研究目的：

评价基于 ACTH 兴奋 AVS 或普通 AVS，判断 PA 患者有无醛固酮单侧优势分泌，进而确定临床治疗方案，并通过治疗后随访，比较两组患者的临床预后。

过程和方法：

本研究通过对有手术意愿的原发性醛固酮增多症患者进行 AVS 来帮助分型诊断，并根据患者的 AVS 结果，确定临床治疗方案，即手术治疗或药物治疗，并通过治疗后随访，比较两组患者的临床预后。如果您同意参与该项目，我们将对每位患者进行编号，并随机分为 ACTH 兴奋组和非兴奋组，并建立病历档案。在研究过程中我们需要收集一些您的临床检测后的剩余血液标本。您的样品标本仅用于本研究。

可能的受益：

国外研究提示与原发性高血压患者相比，PA 患者更早且更易发生心、脑、肾等靶器官损害，随访期间我们会为您开通“绿色通道”，您无需挂号即可进行病情评估和开药，还可

向专家免费咨询与内分泌高血压相关的任何问题。

风险和不适：

我们对您的检查和随访将严格按照现高血压和原醛症诊疗指南和常规进行，不涉及额外的检查和治疗。但因 AVS 操作本身受多种因素影响，国内外报道的成功率为 60%-95%，可能出现插管失败，术中出血、血肿、静脉血栓等相关术中并发症。而疾病本身也可能在随访过程中出现心、脑、肾等靶器官损害及心血管意外。故在研究过程中如有不适，请及时联系我们，以便及时给与处理。

治疗干预方式：

我们将行 AVS 的患者随机分为两组，普通 AVS 组和 ACTH 兴奋 AVS 组。所有操作均严格按照现高血压和原醛症诊疗指南和常规进行。普通 AVS 组术前微量泵入生理盐水 20ml/h，静滴 30min，分别采集右侧和左侧肾上腺静脉血以及相应的外周静脉血，检测皮质醇+醛固酮。ACTH 兴奋 AVS 组术前微量泵入 ACTH 20ml/h，静滴 30min，分别采集右侧和左侧肾上腺静脉血以及相应的外周静脉血，检测皮质醇+醛固酮。我们将根据您的 AVS 结果及其它检查结果决定是否需要手术治疗，开始治疗后您将来院随访，期间需要抽血及登记您的用药信息等。

隐私问题：

如果您决定参加本项目，您的个人资料均属保密。对于您来说，所有的信息将是保密的。

费用及补偿：

您随访过程中的挂号费全免，部分与随访相关的检查如血浆醛固酮和肾素由研究者承担，您需要自行支付除上述意外的常规检查费用及治疗费用。

自由退出：

作为患者，您可随时了解与本项目有关的信息资料和进展，自愿决定（继续）参加还是不（继续）参加，参加后，无论是否发生伤害，或是否严重，您可以选择在任何时候通知责任医生要求退出。您的任何医疗待遇和权益不会因此而受到影响。

联系方式：

如果您有与本研究有关的问题，或您在研究过程中发生了任何不适与损伤，或有关于本项研究参加者权益方面的问题，您可以与您的医生　杨淑敏　联系，电话 15523552235。

试验后利益分享：

当观察结束时，您可以继续联系您的医生就诊。

知情同意签字：

我已经阅读了本知情同意书，并且我的医生已经将此次临床试验的目的、内容、风险和受益情况向我作了详细的解释说明，对我询问的所有问题也给予了解答，我对此项临床研究已经了解，我自愿参加本项研究。

受试者签名：　　　　　　　　　　　　　　　　研究者签名：

日期：　　年　　月　　日　　　　　　　　　　日期：　　年　　月　　日

（注：如果受试者不识字时尚需见证人签名，如果受试者无行为能力时则需代理人同意）

Patient informed consent in English

Adrenal venous sampling with or without adrenocorticotropic hormone stimulation in primary aldosteronism: an outcome-based randomized, double-blinded trial (ADOPA study)

Patients Informed Consent

Introduction of research background:

You are invited to participate in the project "Adrenal venous sampling (AVS) with or without adrenocorticotropic hormone (ACTH) stimulation in primary aldosteronism (PA): an outcome-based randomized, double-blinded trial (ADOPA study)" conducted by the Endocrinology Department of the First Affiliated Hospital of Chongqing Medical University.

The diagnosis of PA is divided into three steps: screening, diagnosis and subtyping. Domestic and foreign guidelines recommend that patients with PA who are willing to undergo surgery should further undergo AVS to determine the dominant side of aldosterone secretion, so as to provide a basis for surgical treatment. At present, AVS is mainly divided into conventional AVS (non-ACTH stimulated AVS) and ACTH stimulated AVS. ACTH stimulated may increasing the rates of successful adrenal vein cannulation, but has been reported to affect the judgment of lateralization.

The aim of this project is to compare the effect of different procedures of AVS (with or without ACTH stimulation) on the long-term outcomes of patients with PA. This informed consent form provides you with some information to help you decide whether to participate in this research. Your participation in this research is voluntary, and this project has approved by the ethics committee of the First Affiliated Hospital of Chongqing Medical University. If you agree to join this project, please see the following instructions:

Research purposes:

To evaluate if the treatment decision based on AVS with or without ACTH stimulation leads to different biochemical and clinical outcomes in patients with PA.

Process and method:

In this study, patients with PA who are willing to undergo surgery were undergo AVS to help

typing diagnosis. The treatment decision based on AVS, including surgical treatment or treated with oral mineralocorticoid receptor antagonists such as spironolactone. The aim is to compare the effect of different procedures of AVS (with or without ACTH stimulation) on the long-term outcomes of patients with PA. If you agree to participate in the project, we will number each patient and randomly divide them into non-ACTH stimulated group and ACTH stimulated group, and establish medical records. During the research, we need to collect some remaining blood samples after your clinical test. The sample is only used for this study.

Possible benefits:

Foreign studies suggest that compared with patients with essential hypertension, PA patients are earlier happened heart, brain, kidney and other target organ damage. During the follow-up period, we will open a "green channel" for you, so that you can evaluate your condition and prescribe drugs without registering, and you can also consult experts for any questions related to endocrine hypertension for free.

Risks and discomfort:

Our examination and follow-up will be carried out in strict accordance with the current guidelines and routine for diagnosis and treatment of hypertension and PA, and no additional examination and treatment will be involved. However, AVS is affected by many factors, the successful cannulation rate reported is 60%-95%, which may lead to cannulation failure, intraoperative bleeding, hematoma, venous thrombosis and other related intraoperative complications. The disease may also cause heart, brain, kidney and other target organ damage and cardiovascular accidents during the follow-up process. Therefore, if you feel uncomfortable during the research, please contact us in time so that you can deal with it in time.

Treatment intervention mode:

We randomly divided the patients into two groups: non-ACTH stimulated group and ACTH stimulated group. All operations were carried out in strict accordance with the current guidelines and routine for the diagnosis and treatment of hypertension and PA. In non-ACTH stimulated AVS, normal saline was administered as continuous infusion which was started 30 minutes before sampling and continued throughout the procedure at 20ml/hr (50μg/hr). Blood samples were collected sequentially from right and left adrenal veins, and blood in the inferior vena cava was collected immediately after the collection of each side of the adrenal vein blood. Cortisol and

aldosterone were measured for each sample. In non-ACTH stimulated AVS, ACTH was administered as continuous infusion which was started 30 minutes before sampling and continued throughout the procedure at 20ml/hr (50μg/hr). Blood samples were collected sequentially from right and left adrenal veins, and blood in the inferior vena cava was collected immediately after the collection of each side of the adrenal vein blood. Cortisol and aldosterone were measured for each sample. We will determine whether surgery is necessary according to your AVS results and other test results. After starting treatment, you will come to the hospital for follow-up visit, during which you will need to draw blood and collect your medication information etc.

Privacy issues:

If you decide to participate in this project, your personal data will be kept confidential. For you, all information will be confidential.

Expenses and compensation:

The registration fee during your follow-up is free, and some follow-up related tests such as plasma aldosterone and renin are fee. You need to pay the routine examination and treatment fees except the above accidents.

Free exit:

You can know the information and progress related to this project at any time, and decide whether to (continue) or not (continue) to participate voluntarily as patients. After participating, you can choose to notify the responsible doctor to ask for withdrawal at any time, regardless of whether there is any injury or not. Any medical rights and interests of you will not be affected.

Contact information:

If you have any questions related to this study, or if you have any discomfort or injury during the study, or if you have any questions about the rights and interests of participants in this study, you can contact your doctor Shumin Yang at telephone number 15523552235.

Benefit sharing after the trial:

When the observation is over, you can continue to contact your doctor.

Informed consent signature:

I have read this informed consent form, and my doctor has explained the purpose, content, risks and benefits of this clinical trial to me in detail, and answered all the questions I asked. I have understood this clinical trial and I voluntarily participated in this trial.

Signature of the subject: signature of the researcher:

Date:

Date:

(Note: If the subject is illiterate, the signature of the witness is required, and if the subject is incompetent, the consent of the agent is required.)

Baseline case report form (CRF)

CONPASS 基线资料收集表

生日：	住院号：	性别：	民族：	职业：	E-mail：
家庭住址：<br>身份证号：			患者电话：<br>家属电话：		加微信：□否，□是<br>座机：
吸烟史：□否/□是；吸烟______年，每日吸烟量 ______支/日；已戒烟_______年<br>饮酒史：□否/□是；饮酒______年，每日饮酒量 ______克/日；（啤酒/白酒，酒精含量：　　　　　　）					
高血压家族史	□无　□父　□母　□兄 □弟　□姐　□妹　□子 □女　若有，请描述其发病年龄、并发症、血压情况				

高血压及其它疾病史

①高血压确诊时间______年_____月；　病程_________；病程中最高血压_________/__________mmHg

②近 2 周用药情况：□无，　□ACEI，　□ARB，　□二氢吡啶 CCB，　□非二氢吡啶 CCB，　□α 受体阻滞剂，　□β 受体阻滞剂，　□

袢利尿剂，　□噻嗪类利尿剂，　□螺内酯，　□依普利酮，　□阿司匹林，　□氯吡格雷，　□硝酸酯类，　□地高辛，　□华法林，　□他汀，　□

其他；以上具体药名、剂量、最后一次用药时间：____

____

③是否有以下症状：□无 □疲劳乏力 □阵发性血压升高 □胸闷、心前区不适 □持续性心悸 □活动后劳累气促 □休息时呼吸困难　□一过性黑矇 □偏侧肢体乏力 □头昏/眩晕 □一过性的失语或失写（若有需记录发作时间、次数、诊治情况）____；

④低血钾病史 □0 无/□1 有；若有，病程_________；最低血钾_______mmol/l，补钾后血钾______mmol/l，前一天补钾量（g/天）：_________________；

⑤夜间打鼾：□无/□有；白天打盹、疲倦：□无/□有；睡眠监测检查：□无/□有；是否诊断为 OSA：□否/□是；

⑥糖尿病：□无/□有；若有，类型：□ 1 型/□ 2 型；病程_________；用降糖药：□无/□有；

⑦冠心病：□无/□有；若有，病程________；既往心肌梗死：□无/□有；既往 PCI 术：□无/□有；既往冠脉搭桥术：□无/□有；既往心绞痛：□无，□稳定性心绞痛　□不稳定性心绞痛；

⑧心衰：□无/□有；NYHA 分级: □Ⅰ级（日常活动量不受限制）　□Ⅱ级（休息时无症状，一般体力活动引起过度疲劳、心悸、气喘或心绞痛）　□Ⅲ级（休息时无症状，但小于一般体力活动即可引起过度疲劳、心悸、气喘或心绞痛）　□Ⅳ级（休息状态下也出现心衰症状）；

⑨房颤：□无，□ 阵发性房颤，□ 持续性房颤；EHRA 分级：□Ⅰ级(无任何症状)；□Ⅱ级(轻微症状，正常的日常活动不受影响)；□Ⅲ级(症状严重，正常的日常活动受到影响)；□Ⅳ级(致残性症状，无法从事日常活动) ；

⑩TIA/脑卒中：□无，□ TIA，□ 脑出血，□ 脑梗死；　慢性阻塞性肺病：□无/□有；　外周血管疾病：□无/□有；

⑪早发冠心病家族史：□无/□有；（早发定义：一级亲属发生冠心病，发病年龄男性<55 岁，女性<65 岁）

其他疾病及其相关治疗____

____

身高(cm)	体重(kg)	腰围(cm)	臀围(cm)	血压 1(mmHg)
血压 2(mmHg)	WBC(*10^9/L)	Hb(g/L)	PLT(*10^9/L)	N(%)
中性粒细胞数(10^9/1)	单核细胞数(10^9/1)	总胆 TBil（umol/1）	结合 DBil（umol/1）	ALB(g/1)
ALT(IU/L)	AST(IU/L)	γ-GGT (U/1)	ALP(ug/1)	BUN(mmol/L)
Cr(μmol/L)	UA(μmol/L)	胱抑素-C(mg/1)	Ca(mmol/L)	Mg(mmol/L)
P(mmol/L)	eGFR ml/(min ╳ 1.73m2)	UACR1(mg/g Cr)	UACR2(mg/g Cr)	TC(mmol/L)

TG(mmol/L)	HDL-c(mmol/L)	LDL-c(mmol/L)	血 K(mmol/L)	血 Na(mmol/L)
血 Cl(mmol/L)	FPG(mmol/L)	PPG(mmol/L)	HbA_1c(%)	hs-CRP (mg/l)
PTH(pg/ml)	$VitB_{12}$(pg/ml)	叶酸(ng/ml)	25 羟 VitD(ng/ml)	同型半胱氨酸 Hcy(mg/l)
MN(ng/l)	NM(ng/l)			

ECG：□未做 □无异常 □房颤/房扑 □3 ST-T 改变 □4 频发早搏 附报告：□有/□无

心脏彩超：□ 未做，□ 无异常 □ 室间隔增厚 □ 左室增大 □ 左房增大 □ 其他： 附报告：□有/□无

颈动脉彩超：□未做，□无异常，□侧斑块：（ ）个，最长径（ ）mm，最宽径（ ）mm ，□右侧斑块：（ ）个，最长径（ ）mm，最宽径（ ）mm ；□ 左侧 IMT 增加：厚度（ ）mm ，□ 右侧 IMT 增加：厚度（ ）mm

ABI：□无/□有，若有，ABI 左： ABI 右： PWV 左（m/s）： PWV 右（m/s）： 附报告：□有/□无

DXA：□未做 □无异常 □骨量减低 □骨质疏松 附报告 □有/□无 | 生活质量评分：□否/□是

24h 尿量（ml）： 尿钠(mmol/24h)： 尿钾(mmol/24h)： 尿醛固酮(ng/24h)： 尿皮质醇(nmol)：
尿肌酐(umol/24h)： 尿标本编号：PA?u 前

肾上腺 CT 结果：□0 无，□1 有，□2 其他检查，具体为：________________________________
左侧： 病变性质：□0 正常；□1 结节；□2 增粗；□3 结节伴增粗 （当出现 1、3 时请填以下信息）
结节个数：______个；结节最大横径_____ mm；结节最大纵径_____ mm；（备注：只填最大结节情况，仅增生不填）
平扫期 CT 值_____，动脉期 CT 值_____，静脉期 CT 值_______（备注：只填最大结节情况）
右侧： 病变性质：□0 正常；□1 结节；□2 增粗；□3 结节伴增粗 （当出现 1、3 时请填以下信息）
结节个数：______个；结节最大横径_____ mm；结节最大纵径_____ mm；（备注：只填最大结节情况，仅增生不填）
平扫期 CT 值____，动脉期 CT 值____，静脉期 CT 值_____（备注：只填最大结节情况）

AVS：□无，□左侧，□右侧，□双侧，□不确定 | 手术方式：□瘤切除 □肾上腺全切
手术：□否/□是 收集组织标本 □否/□是 | 标本多结节：□ 否 □ 是，结节个数__________个

术后并发症：□ 无， □ 肾上腺盐皮质功能减退， □ 肾上腺糖皮质功能减退 | 组织基因检测：□0 无，□1 有

病理：□腺瘤，□增生，□ 增生伴腺瘤，□ 其他，具体为：______________ | 生殖基因检测：□0 无，□1 有

最终诊断：□ APA；□ IHA；□ 皮质醇增多症（临床）；□亚临床库欣；□ 原发高血压；□ 其他，具体为：________________□ 原醛未分型； □ 原醛单侧增生； □ 正常对照； □遗传性原醛症 □嗜铬细胞瘤

手术时间：_____年___ 月____日 | 出院日期：______年___ 月____日 | 预约下次随访时间(1 个月后)：________年 月

出院用药：

检查日期	检查项目	采血	醛固酮 pg/ml	肾素 μIU/ml	皮质醇 nmol/L	ACTH pg/ml	血钾 mmol/L	**编号及数量**
第一次筛查前未停药者，记录所用药：								
**门诊**	立位	8-10am			填节律：8am 16pm 24pm	填节律：8am 16pm 24pm		
	随机（立位）							
	②卡托普利试验	立位			可无	可无		
		10am			可无	可无		
	① 坐位盐水试验	8am			可无	可无		
		12am				可无		

	③氟氢可的松抑制试验	2^{th}血钾：　3^{th}血钾　3^{th}24h 尿量(ml)：　尿钠(mmol/24h)：　尿醛固酮：						
		4^{th}天 7am	可无	可无		可无	可无	
		4^{th}天 10am				可无		
	1mg 地米试验	8am				可无	可无	无

标本收集注意点：基线血浆（p）3 管、血块 2 管、血清（s）分 1-2 管； 收抑制试验试验前后血浆（p）2 管；三个抑制试验代号分别为：①，②，③； 尿标本收集注意点：PA?u 前收 3 管，氟氢可的松抑制试验后 PA?u③收 2 管。

Follow-up CRF

1-2 月随访（V1）

PA 手术以外的患者可电话访视

计划访视时间： 实际访视时间： 电话：

有无核对基线资料是否齐全：□有/□无；（骨密度、ABI、心脏+颈动脉超声、心电图）

目前用药情况：

□无 □ACEI □ARB □钙离子拮抗剂：□二氢吡啶 □非二氢吡啶 □α 受体阻滞剂 □β 受体阻滞剂 □袢利尿剂 □噻嗪类利尿剂 □螺内酯 □依普利酮 □阿司匹林 □氯吡格雷 □硝酸酯类 □地高辛 □华法林 □他汀 □其他；以上具体药名、剂量：

____

____

血压监测情况：□经常小于 140/90mmHg；□经常大于 140/90mmHg（填写血压范围）____________

血压监测频率：□定期；□偶尔；□未监测（嘱至少每周测一次）；休息 10min 监测：□否/□是

螺内酯不良反应：□乳腺增生；□性功能减退；□月经紊乱；□乳房胀痛；□高血钾（大于 5.5mmol/l）；□无；

院外是否仍有低血钾：□未查；□无；□有；若有，次数________血钾水平__________

① 自从上次访视之后是否住过院：□有/□无； 住院时间：
住院原因：

② 是否发生了：a)不稳定型心绞痛：□有/□无；b)稳定型心绞痛：□有/□无；c)新的心肌梗塞：□有/□无；d)新的心衰或心衰恶化：□有/□无； e)TIA 和卒中：□有/□无，□TIA □脑出血□脑梗死；f)心脑血管疾病导致的死亡：□有/□无；

（若发生不稳定型心绞痛、心肌梗塞、心衰、TIA/脑卒中、死亡中的任何一种终点事件，均需填写事件报告表）

____

____

③ 是否有以下症状：□有/□无；□疲劳乏力 □阵发性血压升高 □胸闷、心前区不适 □心悸 □活动后劳累气促 □休息时呼吸困难 □一过性黑矇 □偏侧肢体乏力 □头昏/眩晕 □一过性的失语或失写（若有需记录发作时间、次数、诊治情况）：

身高(cm)	体重(kg)	腰围(cm)	臀围(cm)	
血压$_1$(mmHg)	血压$_2$(mmHg)	UACR(mg/g Cr)		
手术后 1-2 周院外查血日期：	电解质：□未查/□已查：血 K(mmol/L)	血 Na(mmol/L)	血 Cl(mmol/L)	
	肾功能：□未查/□已查：Cr(μmol/L)	BUN(mmol/L)	胱抑素-C(mg/l)	
本次随访	血 K(mmol/L)	血 Na(mmol/L)	血 Cl(mmol/L)	BUN(mmol/L)
Cr(μmol/L)	UA(μmol/L)	Cys-C(mg/L)	Ca(mmol/L)	P(mmol/L)
Mg(mmol/L)	8am 皮质醇 (nmol/l)	8am ACTH(pg/ml)		

卡托普利试验：试验前醛固酮(pg/ml)<br>试验前肾素(μIU/ml)	试验后 2h 醛固酮(pg/ml)<br>试验后 2h 肾素(μIU/ml)
其他检查结果：	
本次随访留血尿标本	生活质量评分：□否/□是；

手术患者疗效判断：

1、临床疗效判断： □ 临床完全缓解；□ 临床部分缓解；□ 临床未缓解：

2、生化疗效判断： □ 生化完全缓解；□ 生化部分缓解；□ 生化未缓解。

诊断：□1 APA；□2 IHA；□3 皮质醇增多症；□4 嗜铬细胞瘤；□5 原发高血压；□6 其他，具体为：__________；□7 原醛未分型；□8 原醛单侧增生； □9 正常对照；□10 GRA

医嘱：

医嘱改变原因：

预约下次访视时间(5 个月后)： 年 月；访视期间如有任何不适或住院，请第一时间通知医生！

留化验单，并顺序粘贴；　　　　　　　　　　　　　　　　　填表医生：

6 月随访（V3）

计划访视时间：　　　　　　　　　　　实际访视时间：　　　　　　　　　　　电话：

目前用药情况：

□无 □ACEI □ARB □钙离子拮抗剂：□二氢吡啶 □非二氢吡啶 □α 受体阻滞剂 □β 受体阻滞剂 □袢利尿剂 □噻嗪类利尿剂 □螺内酯 □依普利酮 □阿司匹林 □氯吡格雷 □硝酸酯类 □地高辛 □华法林 □他汀 □其他；以上具体药名、剂量：____

____

血压监测情况：□经常小于 140/90mmHg；□经常大于 140/90mmHg（填写血压范围）____________________

血压监测频率：□定期；□偶尔；□未监测（嘱至少每周测一次）；休息 10min 监测：□否/□是

螺内酯不良反应：□乳腺增生；□性功能减退；□月经紊乱；□乳房胀痛；□高血钾（大于 5.5mmol/l）；□无；

院外是否仍有低血钾：□未查；□无；□有；若有，次数________血钾水平__________

① 自从上次访视之后是否住过院：□有/□无；　　　　住院时间：

住院原因：

② 是否发生了：a)不稳定型心绞痛：□有/□无；b)稳定型心绞痛：□有/□无；c)新的心肌梗塞：□有/□无；d)新的心衰或心衰恶化：□有/□无；　e)TIA 和卒中：□有/□无，□TIA □脑出血□脑梗死；f)心脑血管疾病导致的死亡：□有/□无；

（若发生不稳定型心绞痛、心肌梗塞、心衰、TIA/脑卒中、死亡中的任何一种终点事件，均需填写事件报告表）

____

____

③ 是否有以下症状：□有/□无；□疲劳乏力 □阵发性血压升高 □胸闷、心前区不适 □心悸 □活动后劳累气促 □休息时呼吸困难 □一过性黑矇 □偏侧肢体乏力 □头昏/眩晕 □一过性的失语或失写（若有需记录发作时间、次数、诊治情况）：

身高(cm)	体重(kg)	腰围(cm)	臀围(cm)	血压 1(mmHg)
Hb(g/L)	WBC(*10^9/L)	N(%)	PLT(*10^9/L)	血压 2(mmHg)
血 K(mmol/L)	血 Na(mmol/L)	血 Cl(mmol/L)	FPG(mmol/L)	HbA_1c(%)
BUN(mmol/L)	Cr(μmol/L)	UA(μmol/L)	Cys-C(mg/L)	Ca(mmol/L)
P(mmol/L)	Mg(mmol/L)	醛固酮(pg/ml)	肾素(μIU/ml)	皮质醇 (nmol/l)
ACTH(pg/ml)	UACR(mg/g Cr)	心酶(必要时)	BNP(必要时)	

其他检查结果：

本次随访不收血尿标本

手术患者疗效判断：

1、临床疗效判断： □ 临床完全缓解；□ 临床部分缓解；□ 临床未缓解：

2、生化疗效判断： □ 生化完全缓解；□ 生化部分缓解；□ 生化未缓解。

诊断：□1 APA；□2 IHA；□3 皮质醇增多症；□4 嗜铬细胞瘤；□5 原发高血压；□6 其他，具体为：____________；□7 原醛未分型；□8 原醛单侧增生； □9 正常对照；□10 GRA

医嘱：

医嘱改变原因：

预约下次访视时间(6 个月后)：　　年　　月；　访视期间如有任何不适或住院，请第一时间通知医生！

留化验单，并顺序粘贴；　　　　　　　　　　　　　　　　填表医生：

1 年随访（V4）

计划访视时间：	实际访视时间：	电话：

目前用药情况：

□无 □ACEI □ARB □钙离子拮抗剂：□二氢吡啶 □非二氢吡啶 □α 受体阻滞剂 □β 受体阻滞剂 □袢利尿剂 □噻嗪类利尿剂 □螺内酯 □依普利酮 □阿司匹林 □氯吡格雷 □硝酸酯类 □地高辛 □华法林 □他汀 □其他；以上具体药名、剂量：

____

____

血压监测情况：□经常小于 140/90mmHg；□经常大于 140/90mmHg（填写血压范围）______________

血压监测频率：□定期；□偶尔；□未监测（嘱至少每周测一次）；休息 10min 监测：□否/□是

螺内酯不良反应：□乳腺增生；□性功能减退；□月经紊乱；□乳房胀痛；□高血钾（大于 5.5mmol/l）；□无；

院外是否仍有低血钾：□未查；□无；□有；若有，次数________血钾水平__________

① 自从上次访视之后是否住过院：□有/□无； 住院时间：

住院原因：

② 是否发生了：a)不稳定型心绞痛：□有/□无；b)稳定型心绞痛：□有/□无；c)新的心肌梗塞：□有/□无；d)新的心衰或心衰恶化：□有/□无； e)TIA 和卒中：□有/□无，□TIA □脑出血□脑梗死；f)心脑血管疾病导致的死亡：□有/□无；

（若发生不稳定型心绞痛、心肌梗塞、心衰、TIA/脑卒中、死亡中的任何一种终点事件，均需填写事件报告表）

____

③ 是否有以下症状：□有/□无；□疲劳乏力 □阵发性血压升高 □胸闷、心前区不适 □心悸 □活动后劳累气促 □休息时呼吸困难 □一过性黑矇 □偏侧肢体乏力 □头昏/眩晕 □一过性的失语或失写（若有需记录发作时间、次数、诊治情况）：

身高(cm)	体重(kg)	腰围(cm)	臀围(cm)	血压 $_1$(mmHg)
Hb(g/L)	WBC($*10^9$/L)	N(%)	PLT($*10^9$/L)	血压 $_2$(mmHg)
血 K(mmol/L)	血 Na(mmol/L)	血 Cl(mmol/L)	FPG(mmol/L)	HbA_1c(%)
TC(mmol/L)	TG(mmol/L)	HDL-c(mmol/L)	LDL-c(mmol/L)	BUN(mmol/L)
Cr(μmol/L)	UA(μmol/L)	Cys-C(mg/L)	Ca(mmol/L)	P(mmol/L)
Mg(mmol/L)	UACR(mg/g Cr)	心酶(必要时)	BNP(必要时)	

ECG：□有/□无， □无异常 □房颤 □ST-T 改变 附报告：□有/□无

心脏彩超：□有/□无 左心室肥厚 □否/□是 附报告：□有/□无

颈动脉彩超：□有/□无 □斑块 □IMT 增厚（厚度: mm）， 附报告：□有/□无

本次随访收血尿标本	生活质量评分：□否/□是

其他检查结果：

手术患者疗效判断：

1、临床疗效判断： □ 临床完全缓解；□ 临床部分缓解；□ 临床未缓解：

2、生化疗效判断： □ 生化完全缓解；□ 生化部分缓解；□ 生化未缓解。

诊断：□1 APA；□2 IHA；□3 皮质醇增多症；□4 嗜铬细胞瘤；□5 原发高血压；□6 其他，具体为：__________；□7 原醛未分型；□8 原醛单侧增生； □9 正常对照；□10 GRA

医嘱：

医嘱改变原因：

预约下次访视时间(1 年后)： 年 月； 访视期间如有任何不适或住院，请第一时间通知医生！

留化验单，并顺序粘贴； 填表医生：

Adrenal vein sampling (AVS) data collecting form

AVS data collection form

住院号		姓名			床号		
AVS 编号		性别			年龄		
PA 编号							
并发症							
项目	时间	醛固酮<br>(pg/ml)	皮质醇<br>(nmol/l)	选择<br>指数	校正后<br>醛固酮	单侧<br>指数	优势侧<br>指数
ACTH 前							
右肾上腺 V1							
右肾上腺 V2							
右肾上腺 V3							
下腔（右 1）							
左肾上腺 AV1							
左肾上腺 AV2							
左肾上腺 CT3							
下腔（左 1）							
ACTH 后							
右肾上腺 V4							
右肾上腺 V5							
右肾上腺 V6							
下腔（右 2）							
左肾上腺 AV4							
左肾上腺 AV5							
左肾上腺 CT6							
下腔（左 2）							
备注：选择指数：肾上腺静脉皮质醇/下腔皮质醇<br>校正后醛固酮：醛固酮/皮质醇<br>单侧指数包括同侧指数与对侧指数<br>同侧指数：（校正后）高侧醛固酮/下腔醛固酮<br>对侧指数：（校正后）低侧醛固酮/下腔醛固酮<br>优势侧指数：（校正后）高侧醛固酮/低侧醛固酮							
判断标准：非 ACTH 兴奋：选择指数≥2.0 提示插管成功<br>ACTH 兴奋：选择指数≥3.0 提示插管成功<br>优势分泌:优势侧指数大于等于 4.0；若优势侧指数 2.0-4.0，需结合 CT 等							
结论：左侧优势() 右侧优势() 双侧分泌() 结合临床() 双侧低于外周()							

报告医生：

时间：

ClinicalTrials.gov Protocol Registration and Results System (PRS) Receipt
Release Date: August 31, 2023

ClinicalTrials.gov ID: NCT04461535

Study Identification

Unique Protocol ID: ADOPA study

Brief Title: Outcomes of AVS With or Without ACTH Stimulation in PA

Official Title: Adrenal Venous Sampling With or Without Adrenocorticotropic Hormone Stimulation in Primary Aldosteronism: an Outcome-based Randomized, Double-blind Trial (ADOPA Study)

Secondary IDs:

Study Status

Record Verification: August 2023

Overall Status: Completed

Study Start: July 8, 2020 [Actual]

Primary Completion: February 20, 2023 [Actual]

Study Completion: February 20, 2023 [Actual]

Sponsor/Collaborators

Sponsor: Chongqing Medical University

Responsible Party: Principal Investigator
Investigator: Qifu Li [qli]
Official Title: Professor.Qifu Li
Affiliation: Chongqing Medical University

Collaborators:

Oversight

U.S. FDA-regulated Drug: No

U.S. FDA-regulated Device: No

U.S. FDA IND/IDE: No

Human Subjects Review: Board Status: Approved
Approval Number: 2020-39-2
Board Name: ethics committee of the first affiliated hospital of Chongqing medical university
Board Affiliation: Chongqing Medical University
Phone: +86-023-89011552
Email: liqifu@yeah.net
Address:

The First Affiliated Hospital of Chongqing Medical University,Chongqing, China

Data Monitoring: No

FDA Regulated Intervention: No

Study Description

Brief Summary: To compare the effect of different procedures of AVS(with or without ACTH stimulation) on the long-term outcomes of patients with PA

Detailed Description: This is a prospective and randomized study involving patients with primary aldosteronism(PA) who completed AVS.

All paticipants will be randomized into ACTH-stimulated group(Intervention group) and ACTH-unstimulated group(Control group) Cannulation was considered successful when the selectivity index (SI), namely plasma cortisol concentration (PCC) in adrenal vein/PCC in IVC≥3 with ACTH stimulation or SI≥2 without ACTH stimulation. The ratio of PAC: PCC on the side with the higher ratio over the contralateral PAC: PCC ratio is defined as the lateralization index (LI). Lateralization of aldosterone excess was defined as LI≥4 irrespective of ACTH use. Patients with LI between 2 and 4 together with contralateral suppression (PAC/PCC of non-dominant side < PAC/PCC of IVC) or CT showing a typical adenoma on the dominant side were also considered to have lateralized disease. Patients with LI < 2 or LI 2-4 without meeting the above criteria were diagnosed as BPA.

In case of technical AVS failure or bilateral PAC/PCC in adrenal venous blood lower than peripheral blood, if the patient meets one of the following criteria, adrenalectomy was recommended: 1) unilateral nodule on CT (≥ 1 cm), no observable nodules or hyperplasia on contralateral adrenal, and PAC ≥ 20 ng/dl, PRC < 5 μIU/ml, K ≤ 3.5mmol/l; 2) unilateral nodule on CT (≥ 1 cm), no observable nodules or hyperplasia on contralateral adrenal, and the contralateral index≤0.5 in AVS.

Surgical intervention is recommended for unilateral PA (UPA) whereas bilateral PA (BPA) is typically treated with oral mineralocorticoid receptor antagonists such as spironolactone. The aim is to compare the long-term outcomes of patients with PA.

Conditions

Conditions: Primary Aldosteronism

Keywords: primary aldosteronism
adrenal venous sampling
adrenocorticotropic hormone

Study Design

Study Type: Interventional

Primary Purpose: Diagnostic

Study Phase: N/A

Interventional Study Model: Parallel Assignment

Number of Arms: 2

Masking:	Single (Participant)
Allocation:	Randomized
Enrollment:	228 [Actual]

Arms and Interventions

Arms	Assigned Interventions
Experimental: AVS with ACTH stimulation<br>Patients divided into AVS with ACTH stimulation group need to undergo stimulation with a continuous cosyntropin infusion.	Drug: Adrenocorticotropic hormone<br>Patients divided into Intervention group need to undergo stimulation with a continuous cosyntropin infusion (50 μg/h started 30 minutes before sampling during AVS). Right and left adrenal venous blood and corresponding peripheral venous blood should be sampled sequentially.<br>Other Names:<br>• ACTH
No Intervention: AVS without ACTH stimulation<br>Patients divided into AVS without ACTH stimulation group take the same procedure of AVS with a continuous saline infusion.	

Outcome Measures

Primary Outcome Measure:

1. Compare the proportion of surgically treated patients with complete biochemical remission in the overall cohort between two groups

   Blood was drawn to measure aldosterone, renin and potassium.According to PASO criteria, outcomes of adrenalectomy for unilateral primary aldosteronism were classified into complete, partial, and absent success, for both clinical and biochemical outcomes.The proportion of complete biochemical remission according to PASO consensus criteria.

   [Time Frame: At 12 months of follow-up.]

Secondary Outcome Measure:

2. Compare the proportion of surgically treated patients who achieved complete clinical remission in the overall cohort between two groups

   The proportion of complete clinical remission according to PASO consensus criteria.Clinical outcomes were determined by the blood pressure response to treatment and the number and dosage of antihypertensive medications.

   [Time Frame: At 12 months of follow-up.]

3. Daily defined doses (DDD) of antihypertensive agents (including MRA), blood pressure, proportion of patients reaching target blood pressure

   Daily defined doses (DDD) of antihypertensive agents (including MRA), blood pressure, proportion of patients reaching target blood pressure in each group irrespective of their treatment after 12 months of follow-up

   [Time Frame: At 12 months of follow-up.]

4. The rate of successful catheterization of bilateral adrenal veins

   Calculate the rate of bilateral successful catheterization.Successful catheterization was defined as SI≥2 without ACTH stimulation or SI≥3 with ACTH stimulation.

   [Time Frame: At baseline.]

5. Adverse events

   Record the occurrence of adverse events, including adrenal venous hemorrhage and related adrenal insufficiency, hypertensive urgencies, anaphylactic shock, venous thrombosis, pulmonary embolism, ect.

[Time Frame: At baseline and 12 month of follow-up.]

Eligibility

Minimum Age: 18 Years

Maximum Age: 70 Years

Sex: All

Gender Based:

Accepts Healthy Volunteers: No

Criteria: 1.Inclusion criteria: Patients who meet the following criterion can be included in this study.

1. Aged between 18-70, male or female, with legal capacity
2. PA diagnosis confirmed by at least one confirmatory test: positive PA screening (ARR≥ 2.0 ng·dl−1/IU·l−1) and at least one positive PA confirmatory test (PAC-post CCT ≥11 ng/dl, PAC-post SSIT≥8·0 ng/dl, or if confirmatory tests were in grey zone (i.e, PAC 80-110 pg/ml two hours after administration of 50 mg captopril or PAC 60-80 pg/ml after the infusion of 2L normal saline), PAC-post FST≥6·0 ng/dl);

2.Exclusion criteria: Patients with one of the following conditions will be excluded in this study:

1. refusal by the patient to undergo AVS or adrenalectomy;
2. meeting the criteria for bypassing AVS [i.e. younger than 35 years old, with typical aldosterone-producing adenomas characteristics (plasma aldosterone >30ng/dl, serum potassium <3·5mmol/l, CT indicated unilateral 1cm low-density adenoma) ;
3. allergic to ACTH or contrast media;
4. pregnant or lactating women;
5. patients with a history of uncontrolled malignant tumor;
6. complicated with Cushing's syndrome [including subclinical Cushing: cortisol after 1mg dexamethasone suppression test (DST)>138 nmol/l or cortisol after 1mg DST 50-138 nmol/l plus adrenocorticotrophic hormone (ACTH)<10pg/ml;
7. diagnosed with familial hyperaldosteronism;
8. with imaging characteristics suggestive of pheochromocytoma or adrenal cortical carcinoma;
9. patients unsuitable for surgery, such as those with heart failure (New York Heart Association (NYHA) class III or IV), severe anemia (Hemoglobin<60g/L), stroke or acute coronary syndrome within 3 months, severe ascites and cirrhosis, estimated glomerulus filtration rate<30ml/min/m2;
10. with alcohol or drug abuse and active mental health disorders.

Contacts/Locations

Central Contact Person: Qifu Li, PhD
Telephone: +86 023-89011552
Email: liqifu@yeah.net

Central Contact Backup:

Study Officials: Qifu Li, PhD
Study Chair

the Chongqing Primary Aldosteronism Study (CONPASS) Group

Locations: **China, Chongqing**
The First Affilated Hospital of Chongqing Medical University
Chongqing, Chongqing, China, 400016
Contact: Qifu Li, M.D., PhD. 023-89011552 liqifu@yeah.net
Contact: Ying Song, M.D. 023-89011552 shuiyunying@126.com

IPDSharing

Plan to Share IPD: No

References

Citations: **[Study Results]** Funder JW, Carey RM, Mantero F, et al. The Management of Primary Aldosteronism: Case Detection, Diagnosis, and Treatment: An Endocrine Society Clinical Practice Guideline[J]. J Clin Endocrinol Metab, 2016, 101(5): 1889-1916.

[Study Results] Nishikawa T, Omura M, Satoh F, et al. Guidelines for the diagnosis and treatment of primary aldosteronism--the Japan Endocrine Society 2009[J]. Endocr J, 2011, 58(9): 711-721.

[Study Results] Deinum J, Groenewoud H, van der Wilt GJ, Lenzini L, Rossi GP. Adrenal venous sampling: cosyntropin stimulation or not?. Eur J Endocrinol. 2019;181(3):D15#D26.

[Study Results] Buffolo F, Monticone S, Williams TA, et al. Subtype Diagnosis of Primary Aldosteronism: Is Adrenal Vein Sampling Always Necessary?. Int J Mol Sci. 2017;18(4):848. Published 2017 Apr 17.

[Study Results] Dekkers T, Prejbisz A, Kool LJS, et al. Adrenal vein sampling versus CT scan to determine treatment in primary aldosteronism: an outcome-based randomised diagnostic trial. Lancet Diabetes Endocrinol. 2016;4(9):739#746.

[Study Results] Williams TA, Lenders JWM, Mulatero P, et al. Outcomes after adrenalectomy for unilateral primary aldosteronism: an international consensus on outcome measures and analysis of remission rates in an international cohort. Lancet Diabetes Endocrinol.

Links:

Available IPD/Information:
