# Supplementary material for: Corticotropin Stimulation in Adrenal Venous Sampling for Patients With Primary Aldosteronism: The ADOPA Randomized Clinical Trial
Source: JAMA Netw Open. 2023 Oct 23;6(10):e2338209. doi: 10.1001/jamanetworkopen.2023.38209 (PMC10594148; doi:10.1001/jamanetworkopen.2023.38209)
Supplement: Supplement 2. — eMethods. Supplemental Methods eTable 1. Proportion of Missing Data in Study Variables eTable 2. Outcomes of Patients in the Trial at 1st and 12th-Month Follow-Up eTable 3. Persistent Hyperaldosteronism in Patients Who Underwent Adrenalectomy eTable 4. Adverse and Serious Adverse Events eTable 5. Baseline Characteristics of the Patients Who Completed Follow-Up eTable 6. Outcomes at 12th-Month Follow-Up in Patients Who Completed Follow-Up eTable 7. Baseline Characteristics of the Patients Excluding Those Whose Treatment Violated the Protocol eTable 8. Baseline Characteristics of the Patients Excluding Those With Failed AVS Plus Bilateral PAC-PCC Ratio in Adrenal Venous Blood Lower Than Peripheral Blood eTable 9. Secondary Outcomes at 12th-Month Follow-Up in Patients Excluding Those Whose Treatment Violated the Protocol eTable 10. Secondary Outcomes at 12th-Month Follow-Up in Patients Excluding Those With Failed AVS Plus Bilateral PAC-PCC Ratio in Adrenal Venous Blood Lower Than Peripheral Blood eTable 11. Baseline Characteristics of the Patients Excluding Those With LI 2 to 4 eTable 12. Outcomes at 12th-Month Follow-Up in Patients Excluding Those With LI 2 to 4 eTable 13. Outcomes at 12th-Month Follow-Up With Increased Selectivity Index eTable 14. Outcomes at 12th-Month Follow-Up With Decreased LI eReferences [file jamanetwopen-e2338209-s002.pdf]

Supplementary Online Content

Yang S, Du Z, Zhang X, et al; Chongqing Primary Aldosteronism Study (CONPASS) Group. Corticotropin stimulation in adrenal venous sampling for patients with primary aldosteronism: the ADOPA randomized clinical trial. *JAMA Netw Open*. 2023;6(10):e2338209. doi:10.1001/jamanetworkopen.2023.38209

eMethods. Supplemental Methods
eTable 1. Proportion of Missing Data in Study Variables
eTable 2. Outcomes of Patients in the Trial at 1st and 12th-Month Follow-Up
eTable 3. Persistent Hyperaldosteronism in Patients Who Underwent Adrenalectomy
eTable 4. Adverse and Serious Adverse Events
eTable 5. Baseline Characteristics of the Patients Who Completed Follow-Up
eTable 6. Outcomes at 12th-Month Follow-Up in Patients Who Completed Follow-Up
eTable 7. Baseline Characteristics of the Patients Excluding Those Whose Treatment Violated the Protocol
eTable 8. Baseline Characteristics of the Patients Excluding Those With Failed AVS Plus Bilateral PAC-PCC Ratio in Adrenal Venous Blood Lower Than Peripheral Blood
eTable 9. Secondary Outcomes at 12th-Month Follow-Up in Patients Excluding Those Whose Treatment Violated the Protocol
eTable 10. Secondary Outcomes at 12th-Month Follow-Up in Patients Excluding Those With Failed AVS Plus Bilateral PAC-PCC Ratio in Adrenal Venous Blood Lower Than Peripheral Blood
eTable 11. Baseline Characteristics of the Patients Excluding Those With LI 2 to 4
eTable 12. Outcomes at 12th-Month Follow-Up in Patients Excluding Those With LI 2 to 4
eTable 13. Outcomes at 12th-Month Follow-Up With Increased Selectivity Index
eTable 14. Outcomes at 12th-Month Follow-Up With Decreased LI
eReferences

This supplementary material has been provided by the authors to give readers additional information about their work.

© 2023 Yang S et al. *JAMA Network Open*.

eMethods. Supplemental methods

Inclusion and exclusion criteria

The inclusion criteria were: aged between 18-70 years and PA diagnosis confirmed by at least one confirmatory test. Exclusion criteria were: 1) refusal by the patient to undergo AVS or adrenalectomy; 2) meeting the criteria for bypassing AVS [i.e. younger than 35 years old, with typical aldosterone-producing adenomas characteristics (plasma aldosterone >30ng/dL, serum potassium ≤3.5mEq/L, CT indicated unilateral 1cm low-density adenoma)[1]; 3) allergic to ACTH or contrast media; 4) pregnant or lactating women; 5) patients with a history of uncontrolled malignant tumor; 6) complicated with Cushing's syndrome [including subclinical Cushing: cortisol after 1mg dexamethasone suppression test (DST)>5μg/dL or cortisol after 1mg DST 1.8-5μg/dL plus adrenocorticotrophic hormone (ACTH)<10 pg/mL[2]; 7) diagnosed with familial hyperaldosteronism; 8) with imaging characteristics suggestive of pheochromocytoma or adrenal cortical carcinoma; 9) patients unsuitable for surgery, such as those with heart failure (New York Heart Association (NYHA) class III or IV), severe anemia (Hemoglobin<60g/L), stroke or acute coronary syndrome within 3 months, severe ascites and cirrhosis, estimated glomerulus filtration rate<30ml/min/m^2; 10) with alcohol or drug abuse and active mental health disorders. The ethics committee of the First Affiliated Hospital of Chongqing Medical University approved the protocol. Informed written consent was obtained from each participant.

Diagnosis of PA

Detailed screening methods

Before screening, diuretic therapy, including mineralocorticoid receptor antagonists (MRA), was withdrawn for at least 4 weeks, and angiotensin-converting enzyme inhibitors, angiotensin-II receptor blockers and β-blockers were stopped for at least two weeks. Non-dihydropyridine calcium channel blockers and/or α-adrenergic blockers were allowed for uncontrolled hypertension. Samples for plasma renin concentration (PRC) and plasma aldosterone concentration (PAC) were collected in the morning after participants had been out of bed for at least 2 hours and after they had been seated for 15 minutes.

Confirmatory test criteria

PA was confirmed if at least one of the following criteria was met: 1) PAC ⩾11 ng/dL two hours after administration of 50 mg captopril[3]; 2) PAC ⩾8.0 ng/dL after the infusion of 2L normal saline[4]; 3) if confirmatory tests were in grey zone (i.e, PAC 8-11 ng/dL two hours after administration of 50 mg captopril or PAC 6-8 ng/dL after the infusion of 2L normal saline), we further performed fludrocortisone suppression test and diagnosed as PA if PAC on the fourth day of fludrocortisone administration exceeded 6.0 ng/dL[1].

Adrenal CT criteria

The CT findings were classified into unilateral lesion, bilaterally normal, and bilateral lesions. Adrenal lesion included nodule (defined as round or oval, with smooth margins, well defined, ⩾4mm in diameter) and hyperplasia (if adrenal gland thickness measured ⩾10 mm in diameter)[5]. If no nodule or hyperplasia was found, the CT result were considered as normal.

Randomization methods

Both the patients and AVS performers were masked to the allocation. An investigator (J. H) was

© 2023 Yang S et al. *JAMA Network Open*.

responsible for the randomization, using R software (version 4.0.3) to generate random number table and preparing ACTH or normal saline according to the randomization result just before the initiation of AVS. In details, we used the random function of "runif" to generate random numbers based on the pre-set sample size. We assigned ranks to these generated random numbers with the "rank" function. We obtained a random grouping table after dividing the ranked random numbers into two groups with a ratio of 1:1. We generated allocation concealment sequence scheme and prepared ACTH or normal saline according to the randomization result just before the initiation of AVS. The allocation sequence scheme was generated without any stratification.

AVS procedures

Normal saline or ACTH was administered as continuous infusion which was started 30 minutes before sampling and continued throughout the procedure at 20ml/hr (50μg/hr). Blood samples were collected sequentially from right and left adrenal veins. Three tubes of blood (2ml for one tube) in each adrenal vein were collected consecutively, and one tube of blood in the inferior vena cava (IVC) was collected immediately after the collection of each side of the adrenal vein blood. The average results of three adrenal vein blood samples were used for index calculation. Cortisol and aldosterone were measured in each sample and ACTH in the IVC sample was also measured.

In this study, the patients and AVS performers were blinded. Due to AVS results, such as peripheral cortisol concentration, selectivity index, etc. may reveal the grouping, therefore, the investigators analyzing AVS results were not blinded. Two endocrinologists (Q.L and Y.S) decided on interpretation of AVS results.

Calculation of daily defined doses (DDD)

The DDD was calculated according to the ATC/DDD Index (https://www.whocc.no/atc_). For instance, 75mg of spirolactone has a daily defined dose of 1.0, as does 5 mg of amlodipine.

Sample size calculation

The trial was designed to have a power of 80% to detect a difference between the two groups in the proportion of complete biochemical remission of 19%, based on the following assumptions: compared with AVS without ACTH stimulation, AVS with ACTH stimulation reduced the number of unsuccessful cannulations in patients with PA by 28%[6].Based on our previous research[3], 70% of the patients in our center underwent AVS was UPA, and 93% UPA achieved complete biochemical success post-adrenalectomy. We assumed that, successful cannulation rate was 70% in AVS without ACTH stimulation, and the complete biochemical success rate would be 0.7*0.7*0.93=0.45 in AVS without ACTH stimulation and 0.7*(0.7+0.28)*0.93= 0.64 in AVS with ACTH stimulation. Calculation of the sample size, with a two-sided significance level of 0.05, indicates a required total sample size of 208 patients, 104 in each group, having a power of 80% to detect a difference between the two groups in the proportion of complete biochemical remission of 19%. To account for a ~10 % drop out rate we aimed at a sample size of 115 patients.

Missing data assessment

The extent of missing data of study variables have been added in eTable1. Missing data were imputed with the multivariate imputation by chained equations algorithm. Regression models were utilized to predict the missing values.

© 2023 Yang S et al. *JAMA Network Open*.

eTable 1. Proportion of Missing Data in Study Variables

	non-ACTH stimulated(n=115)	ACTH stimulated(n=113)
Age, %	0	0
Sex, %	0	0
BMI, %	0	0
DDD, %	0	0
SBP, %	0	0
DBP, %	0	0
Serum K^+, %	0	0
Serum Na^+, %	0	0
eGFR, %	0	0
Upright PAC, %	0	0
Upright PRC, %	0	0
Post-CCT PAC, %	0	0
Post-CCT PRC, %	0	0
PAC in left AV, %	0	0
PAC in right AV, %	1	3
PCC in left AV, %	0	0
PCC in right AV, %	1	3
PAC/PCC in left AV, %	0	0
PAC/PCC in right AV, %	1	3
PAC in IVC1, %	0	1
PAC in IVC2, %	0	1
PCC in IVC1, %	0	1

© 2023 Yang S et al. *JAMA Network Open*.

PCC in IVC2, %	0	1
PAC/PCC in IVC1, %	0	1
PAC/PCC in IVC2, %	0	1
Lateralization index	8	7
Selectivity index in left AV	0	0
Selectivity index in right AV	1	3
DDD at 1stmonth, %	7	6
SBP at 1stmonth, %	7	6
DBP at 1stmonth, %	7	6
Serum K^+ at 1stmonth, %	7	6
eGFR at 1stmonth, %	7	6
PAC at 1stmonth, %	7	6
PRC at 1stmonth, %	7	6
DDD at 12thmonth, %	13	15
SBP at 12thmonth, %	7	6
DBP at 12thmonth, %	7	6
Serum K^+ at 12thmonth, %	7	6
eGFR at 12thmonth, %	7	6
PAC at 12thmonth, %	7	6
PRC at 12thmonth, %	7	6
Adverse events, %	0	0

BMI: body mass index; SBP: systolic blood pressure; DBP: diastolic blood pressure; eGFR: estimated glomerular filtration rate; PAC: plasma aldosterone concentration; PRC: plasma renin concentration; CCT: captopril challenge test; PCC: plasma cortisol concentration; AV: adrenal vein; IVC: inferior vena cava; IVC1: IVC blood collected immediately after the collection of left adrenal vein blood; IVC2: IVC blood collected immediately after the collection of right adrenal vein blood; DDD: daily defined dose.

© 2023 Yang S et al. *JAMA Network Open*.

eTable 2. Outcomes of patients in trial at 1st and 12th month follow-up

	Non-ACTH stimulated (n=115)						ACTH stimulated (n=113)					
	Adrenalectomy(n=68)			Mineralocorticoid receptor antagonist(n=47)			Adrenalectomy(n=65)			Mineralocorticoid receptor antagonist(n=48)		
	1st month	12th month	P value	1st month	12th month	P value	1st month	12th month	P value	1st month	12th month	P value
Daily defined doses	0(0,1.0)	0(0,1.0)	0.78	1.5(1.0,2.3)	1.6(1.0,2.3)	0.78	0(0,0.4)	0(0,0.1)	0.70	1.4(0.8,2.3)	1.5(0.8,2.3)	0.85
SBP (mmHg)	132±18	127±14	0.11	136±18	130±13	0.05	133±21	129±19	0.24	140±19	133±16	0.07
DBP (mmHg)	87±12	85±11	0.28	90±13	85±10	0.06	87±13	87±14	0.79	91±15	87±12	0.17
Serum K^+ (mEq/L)†	4.4(4.1,4.6)	4.3(4.1,4.5)	0.24	4.1(3.8,4.3)	4.2(3.8,4.4)	0.78	4.4(4.2,4.7)	4.3(4.2,4.5)	0.17	4.1(3.8,4.3)	4.2(3.8,4.5)	0.22
eGFR (ml/(min·1.73m^2)	73.6(64.0,94.6)	83.5(63.6,94.0)	0.42	80.6(65.2,90.5)	83.1(72.3,97.4)	0.19	70.5(62.5,84.1)	76.1(61.7,88.4)	0.36	80.8(69.4,104.0)	84.4(72.7,98.7)	0.62
PAC (ng/dL) ‡	6.8(3.5,10.4)	8.9(4.9,11.4)	0.04	22.3(12.4,31.3)	16.7(10.5,23.6)	0.15	5.4(3.5,9.4)	6.8(4.7,10.6)	0.04	21.0(14.6,30.3)	19.8(15.0,24.3)	0.17
PRC (pg/mL)§	7.6(3.0,14.5)	15.4(4.1,26.1)	0.02	7.6(3.0,18.4)	10.2(4.6,28.8)	0.14	8.1(2.9,14.5)	13.5(6.1,28.8)	0.002	9.8(5.3,18.9)	11.6(3.3,25.0)	0.70

Data were expressed as %, median (interquartile range), or mean ± SD. SBP: systolic blood pressure; DBP: diastolic blood pressure; eGFR: estimated glomerular filtration rate; PAC: plasma aldosterone concentration; PRC: plasma renin concentration; P values represent the comparison between the 1st month follow-up and the 12th month follow-up.

†: To convert mEq/L to mmol/L, multiply by 1.

‡: To convert ng/dL to pmol/L, multiply by 27.74.

§: To convert pg/mL to pmol/L, multiply by 0.0237.

© 2023 Yang S et al. *JAMA Network Open*.

eTable 3. Persistent hyperaldosteronism in patients who underwent adrenalectomy

Patient number	group	Sex	Age (y)	SBP mmHg	DBP mmHg	Serum K^+ mEq/L†	Adrenal CT	PAC ng/dL‡	PRC pg/mL§	Post-SIT PAC ng/dL‡	Post-SIT PRC pg/mL§	Post-CCT PAC ng/dL‡	Post-CCT PRC pg/mL§
Baseline													
1	ACTH-stimulated	Female	65	142	91	3.2	Left nodule	62.4	0.3	28.8	0.5	32.0	0.3
2	ACTH-stimulated	Male	64	206	98	2.8	Bilateral normal	29.0	0.4	14.5	0.3	24.9	0.4
3	non-ACTH-stimulated	Male	37	156	102	3.6	Left hyperplasia	27.3	0.9	13.7	1.2	19.4	1.0
Follow-up													
1*	ACTH-stimulated	Female	65	123	83	4.4	--	12.6	0.8	--	--	--	--
2*	ACTH-stimulated	Male	64	177	95	3.7	--	13.4	1.7	--	--	--	--
3	non-ACTH-stimulated	Male	37	115	82	4.0	--	23.3	3.0	--	--	16.4	2.5

*: The patient refused to take captopril challenge test at follow-up.

SBP: systolic blood pressure; DBP: diastolic blood pressure; PAC: plasma aldosterone concentration; PRC: plasma renin concentration; SIT: saline infusion test; CCT: captopril challenge test.

†: To convert mEq/L to mmol/L, multiply by 1.

‡: To convert ng/dL to pmol/L, multiply by 27.74.

§: To convert pg/mL to pmol/L, multiply by 0.0237.

© 2023 Yang S et al. *JAMA Network Open*.

eTable 4. Adverse and Serious Adverse Events

	non-ACTH stimulated (n=115)	ACTH stimulated (n=113)
Diagnostic phase		
SAE	Stroke (n=1); Respiratory failure (n=1).	Respiratory failure (n=1); Femoral vein thrombosis after AVS (n=1).
AE	Nausea and mild headache during fludrocortisone-suppression test (n=2); Abdominal discomfort during fludrocortisone-suppression test (n=1); Adrenal vein rupture during adrenal vein blood sampling (n=2); Abdominal discomfort after taking potassium chloride (n=1); Postural hypotension after taking doxazosin (n=1); Headache and dizziness after taking doxazosin (n=1); Transient angina (n=1); Bleeding at puncture site of femoral vein (n=2).	Nausea during fludrocortisone-suppression test (n=1); Allergic reaction to fludrocortisone (n=1); Acute upper respiratory infection during fludrocortisone-suppression test (n=1); Headache and dizziness after taking doxazosin (n=1); Hypertensive urgencies during saline infusion test (n=2); Adrenal vein rupture during adrenal vein blood sampling (n=2); Postural hypotension after taking doxazosin (n=1); Splenic artery aneurysms (n=1).
Adrenalectomy		
SAE	Stroke (n=1); Persistent post-operative hypokalemia (n=1);	--
AE	Transient post-operative hypokalemia (n=2); Anesthetic drug-induced liver injury (n=1); Anesthetic drug-induced vomiting (n=2); Post-operative pneumonia (n=2); Transient post-operative angina (n=1); Transient post-operative tachycardia (n=1); Prolonged postoperative ileus (n=8); Post-operative adrenal insufficiency (n=1); Post-operative intermuscular vein thrombosis of left lower extremity (n=1); Post-operative intermuscular vein thrombosis of right lower extremity (n=1); Post-operative hemoglobin decreased progressively (n=1).	Anesthetic drug-induced liver injury (n=2); Anesthetic drug-induced vomiting (n=2); Prolonged postoperative ileus(n=8); Post-operative renal insufficiency (n=1); Post-operative urinary tract infection (n=1); Post-operative adrenal insufficiency (n=1); Postural hypotension(n=3).
Follow-up		
SAE	--	--
AE	Spironolactone side effects(n=6).	Spironolactone side effects(n=5); Percutaneous coronary intervention for coronary heart disease (n=1); Hysterectomy due

© 2023 Yang S et al. *JAMA Network Open*.

		to uterine leiomyomas (n=1); Aortic arch aneurysm (n=1).
Total	42 (SAE:4; AE:38)	38 (SAE:2; AE:36)

© 2023 Yang S et al. *JAMA Network Open*.

eTable 5. Baseline characteristics of the patients who completed follow-up

Characteristic	non-ACTH stimulated (n=107)	ACTH stimulated (n=106)
Age (y)	50.0(41.0,57.0)	50.5(42.8,57.0)
Sex		
Male, n (%)	65(60.7)	58(54.7)
Female, n (%)	42(39.3)	48(45.3)
BMI (kg/m^2)	24.3(22.9,26.7)	24.9(22.2,27.3)
Daily defined dose	1.3(0.9,2.0)	1.1(1.0,2.0)
SBP (mmHg)	148±18	153±17
DBP (mmHg)	92±14	94±12
Serum K^+ (mEq/L)†	3.6(3.3,3.9)	3.4(3.1,3.8)
Serum Na^+ (mEq/L)†	142.0(140.0,143.0)	141.0(140.0,143.0)
eGFR (ml/(min·1.73m^2))	88.5(77.9,107.0)	88.8(77.0,108.0)
Upright PAC (ng/dL) ‡	21.2(14.9,31.0)	22.8(18.9,31.0)
Upright PRC (pg/mL)§	1.9(0.7,4.2)	1.6(0.6,3.0)
Post-CCT PAC (ng/dL) ‡	19.3(13.4,29.1)	19.4(15.4,26.8)
Post-CCT PRC (pg/mL)§	2.1(0.8,6.8)	2.3(0.8,5.3)

Data were expressed as %, median (interquartile range), or mean ± SD. BMI: body mass index; SBP: systolic blood pressure; DBP: diastolic blood pressure; eGFR: estimated glomerular filtration rate; PAC: plasma aldosterone concentration; PRC: plasma renin concentration; CCT: captopril challenge test.

†: To convert mEq/L to mmol/L, multiply by 1.

‡: To convert ng/dL to pmol/L, multiply by 27.74.

§: To convert pg/mL to pmol/L, multiply by 0.0237.

© 2023 Yang S et al. *JAMA Network Open*.

eTable 6. Outcomes at 12th month follow-up in patients who completed follow-up

	Total (n=213)			Adrenalectomy(n=118)			Mineralocorticoid receptor antagonist(n=95)		
	non-ACTH stimulated (n=107)	ACTH stimulated (n=106)	P value	non-ACTH stimulated (n=60)	ACTH stimulated (n=58)	P value	non-ACTH stimulated (n=47)	ACTH stimulated (n=48)	P value
Complete biochemical remission, n (%)	59(55.1)	56(52.8)	0.74	59(98.3)	56(96.6)	0.54	NA	NA	NA
Complete clinical remission, n (%)	26(24.3)	31(29.2)	0.42	26(43.3)	31(53.4)	0.27	NA	NA	NA
Defined daily dose	1.0(0,1.6)	0.6(0,2.0)	0.35	0(0,1.0)	0(0,0.1)	0.10	1.6(1.0,2.3)	1.5(0.8,2.3)	0.75
Adverse events, n (%)	36(33.6)	38(35.8)	0.74	26(43.3)	25(43.1)	0.98	10(21.3)	13(27.1)	0.51
SBP (mmHg)	128±13	131±18	0.26	127±14	129±19	0.62	130±13	133±16	0.25
DBP (mmHg)	85±10	87±13	0.24	85±11	87±14	0.34	85±10	87±12	0.50
Achieving target blood pressure, n (%)	78(72.9)	69(65.1)	0.28	47(78.3)	42(72.4)	0.59	31(66.0)	27(56.2)	0.45

Data were expressed as %, median (interquartile range), or mean ± SD. SBP: systolic blood pressure; DBP: diastolic blood pressure; PAC: plasma aldosterone concentration; PRC: plasma renin concentration. P values represent the comparison between non-ACTH stimulated and ACTH stimulated.

© 2023 Yang S et al. *JAMA Network Open*.

eTable 7. Baseline characteristics of the patients excluding those whose treatment violated the protocol*

Characteristic	non-ACTH stimulated (n=109)	ACTH stimulated (n=107)
Age (y)	49.0(40.5,56.5)	50.0(43.0,57.0)
Sex		
Male, n (%)	66(60.6)	60(56.1)
Female, n (%)	43(39.4)	47(43.9)
BMI (kg/m^2)	24.3(22.9,26.9)	24.8(22.0,27.1)
Daily defined dose	1.3(1.0,2.0)	1.3(1.0,2.0)
SBP (mmHg)	150±17	152±17
DBP (mmHg)	93±13	93±12
Serum K^+ (mEq/L)†	3.6(3.2,3.8)	3.4(3.1,3.8)
Serum Na^+ (mEq/L)†	142.0(140.0,144.0)	141.0(140.0,143.0)
eGFR (ml/(min·1.73m^2))	88.5(77.6,107.0)	88.2(76.5,107.0)
Upright PAC (ng/dL) ‡	21.8(15.5,31.2)	23.3(19.2,34.8)
Upright PRC (pg/mL)§	1.8(0.7,4.2)	1.8(0.6,3.4)
Post-CCT PAC (ng/dL) ‡	19.6(13.5,29.1)	19.7(15.8,28.8)
Post-CCT PRC (pg/mL)§	1.9(0.7,6.8)	2.4(0.9,5.4)

***** Patients whose treatment violated the protocol were excluded, including the patients diagnosed as UPA by AVS but treated with medication (five in non-ACTH and five in ACTH stimulated group) and the patients did not meet surgery criteria but treated with adrenalectomy (one each group respectively).

Data were expressed as %, median (interquartile range), or mean ± SD. BMI: body mass index; SBP: systolic blood pressure; DBP: diastolic blood pressure; eGFR： estimated glomerular filtration rate; PAC: plasma aldosterone concentration; PRC: plasma renin concentration; CCT: captopril challenge test.

P values represent the comparison between non-ACTH stimulated and ACTH stimulated.

†: To convert mEq/L to mmol/L, multiply by 1.

‡: To convert ng/dL to pmol/L, multiply by 27.74.

§: To convert pg/mL to pmol/L, multiply by 0.0237.

© 2023 Yang S et al. *JAMA Network Open*.

eTable 8. Baseline characteristics of the patients excluding those with failed AVS plus bilateral PAC/PCC in adrenal venous blood lower than peripheral blood*

Characteristic	non-ACTH stimulated (n=101)	ACTH stimulated (n=103)
Age (y)	49.0(39.5,56.5)	49.0(43.0,55.0)
Sex		
Male, n (%)	64(63.4)	60(58.3)
Female, n (%)	37(36.6)	43(41.7)
BMI (kg/m^2)	24.2(22.8,26.2)	24.9(22.1,27.1)
Daily defined dose	1.3(0.8,2.0)	1.3(1.0,2.0)
SBP (mmHg)	149±17	153±18
DBP (mmHg)	92±14	94±13
Serum K^+ (mEq/L)†	3.6(3.3,3.8)	3.4(3.1,3.8)
Serum Na^+ (mEq/L)†	142.0(140.0,144.0)	142.0(140.0,143.0)
eGFR (ml/(min·1.73m^2))	88.6(78.3,108.0)	88.0(77.2,108.0)
Upright PAC (ng/dL) ‡	21.8(15.4,31.7)	23.1(19.2,31.5)
Upright PRC (pg/mL)§	1.5(0.6,3.9)	1.9(0.7,3.6)
Post-CCT PAC (ng/dL) ‡	19.8(14.1,29.1)	19.6(15.8,26.7)
Post-CCT PRC (pg/mL)§	1.6(0.6,6.1)	2.4(0.9,5.3)

* Patients with failed AVS (nine in non-ACTH and eight in ACTH stimulated group) plus bilateral PAC/PCC in adrenal venous blood lower than peripheral blood (five in non-ACTH and two in ACTH stimulated group) were excluded.

Data were expressed as %, median (interquartile range), or mean ± SD. BMI: body mass index; SBP: systolic blood pressure; DBP: diastolic blood pressure; eGFR: estimated glomerular filtration rate; PAC: plasma aldosterone concentration; PRC: plasma renin concentration; CCT: captopril challenge test; PCC: plasma cortisol concentration. P values represent the comparison between non-ACTH stimulated and ACTH stimulated.

†: To convert mEq/L to mmol/L, multiply by 1.

‡: To convert ng/dL to pmol/L, multiply by 27.74.

© 2023 Yang S et al. *JAMA Network Open*.

§: To convert pg/mL to pmol/L, multiply by 0.0237.

© 2023 Yang S et al. *JAMA Network Open*.

eTable 9. Secondary outcomes at 12th month follow-up in patients excluding those whose treatment violated the protocol*

	Total (n=216)			Adrenalectomy(n=131)			Mineralocorticoid receptor antagonist(n=85)		
	non-ACTH stimulated (n=109)	ACTH stimulated (n=107)	P value	non-ACTH stimulated (n=67)	ACTH stimulated (n=64)	P value	non-ACTH stimulated (n=42)	ACTH stimulated (n=43)	P value
Daily defined doses	1.0(0,1.7)	0.6(0,2.0)	0.31	0(0,1.0)	0(0,0.1)	0.10	1.7(1.1,2.3)	1.5(0.9,2.3)	0.60
Adverse events, n (%)	40(36.7)	34(31.8)	0.45	31(46.3)	24(37.5)	0.31	9(21.4)	10(23.3)	0.84
Achieving target blood pressure, n (%)	74(67.9)	68(63.6)	0.50	46(68.7)	42(65.6)	0.71	28(66.7)	26(60.5)	0.55
SBP (mmHg)	129±13	130±17	0.58	128±14	128±16	0.10	131±13	133±17	0.41
DBP (mmHg)	85±10	87±12	0.42	85±11	87±13	0.57	85±10	87±12	0.56

* Patients whose treatment violated the protocol were excluded, including the patients diagnosed as UPA by AVS but treated with medication (five in non-ACTH and five in ACTH stimulated group) and the patients did not meet surgery criteria but treated with adrenalectomy (one each group respectively).

Data were expressed as %, median (interquartile range), or mean ± SD. SBP: systolic blood pressure; DBP: diastolic blood pressure; NA: not applicable. P values represent the comparison between non-ACTH stimulated and ACTH stimulated.

© 2023 Yang S et al. *JAMA Network Open*.

eTable 10. Secondary outcomes at 12th month follow-up in patients excluding those with failed AVS plus bilateral PAC/PCC in adrenal venous blood lower than peripheral blood*

	Total (n=204)			Adrenalectomy(n=124)			Mineralocorticoid receptor antagonist(n=80)		
	**non-ACTH stimulated (n=101)**	**ACTH stimulated (n=103)**	**P value**	**non-ACTH stimulated (n=65)**	**ACTH stimulated (n=59)**	**P value**	**non-ACTH stimulated (n=36)**	**ACTH stimulated (n=44)**	**P value**
Daily defined doses	0.9(0,1.5)	0.6(0,2.0)	0.78	0(0,1.0)	0(0,0.2)	0.15	1.5(0.9,2.3)	1.5(0.8,2.3)	0.86
Adverse events, n (%)	38(37.6)	35(34.0)	0.59	31(47.7)	23(39.0)	0.33	7(19.4)	12(27.3)	0.41
Achieving target blood pressure, n (%)	70(69.3)	64(62.1)	0.28	45(69.2)	38(64.4)	0.57	25(69.4)	26(59.1)	0.34
SBP (mmHg)	127±14	130±17	0.19	127±14	128±16	0.78	128±13	133±17	0.12
DBP (mmHg)	85±11	87±12	0.23	85±11	87±13	0.39	85±10	87±12	0.41

* Patients with failed AVS (nine in non-ACTH and eight in ACTH stimulated group) plus bilateral PAC/PCC in adrenal venous blood lower than peripheral blood (five in non-ACTH and two in ACTH stimulated group) were excluded.

Data were expressed as %, median (interquartile range), or mean ± SD. SBP: systolic blood pressure; DBP: diastolic blood pressure; NA: not applicable. P values represent the comparison between non-ACTH stimulated and ACTH stimulated.

© 2023 Yang S et al. *JAMA Network Open*.

eTable 11. Baseline characteristics of the patients excluding those with LI 2-4*

Characteristic	non-ACTH stimulated (n=98)	ACTH stimulated (n=97)
Age (y)	50.5(41.0,57.0)	50.0(43.5,57.0)
Sex		
Male, n (%)	60(61.2)	53(54.6)
Female, n (%)	38(38.8)	44(45.4)
BMI (kg/m^2)	24.2(22.8,26.2)	24.7(22.1,27.1)
Daily defined dose	1.3(1.0,2.0)	1.0(1.0,2.0)
SBP (mmHg)	149±18	151±18
DBP (mmHg)	92±14	93±12
Serum K^+ (mEq/L)†	3.6(3.2,3.8)	3.4(3.1,3.8)
Serum Na^+ (mEq/L)†	142.0(140,144.0)	142.0(140.0,143.0)
eGFR (ml/(min·1.73m^2))	88.6(78.2,107.0)	88.0(76.9,108.0)
Upright PAC (ng/dL) ‡	22.8(15.5,33.5)	23.3(19.2,34.7)
Upright PRC (pg/mL)§	1.9(0.7,4.2)	1.5(0.5,2.7)
Post-CCT PAC (ng/dL) ‡	20.3(13.8,31.4)	19.9(16.1,30.4)
Post-CCT PRC (pg/mL)§	1.8(0.6,6.7)	1.9(0.7,4.1)

***** Patients with LI 2-4 were excluded (seventeen in non-ACTH and sixteen in ACTH stimulated group).

Data were expressed as %, median (interquartile range), or mean ± SD. BMI: body mass index; SBP: systolic blood pressure; DBP: diastolic blood pressure; eGFR: estimated glomerular filtration rate; PAC: plasma aldosterone concentration; PRC: plasma renin concentration; CCT: captopril challenge test.

P values represent the comparison between non-ACTH stimulated and ACTH stimulated.

†: To convert mEq/L to mmol/L, multiply by 1.

‡: To convert ng/dL to pmol/L, multiply by 27.74.

§: To convert pg/mL to pmol/L, multiply by 0.0237.

© 2023 Yang S et al. *JAMA Network Open*.

eTable 12. Outcomes at 12th month follow-up in patients excluding those with LI 2-4*

	Total (n=195)			Adrenalectomy(n=124)			Mineralocorticoid receptor antagonist(n=71)		
	non-ACTH stimulated (n=98)	ACTH stimulated (n=97)	P value	non-ACTH stimulated (n=63)	ACTH stimulated (n=61)	P value	non-ACTH stimulated (n=35)	ACTH stimulated (n=36)	P value
Complete biochemical remission, n (%)	55(56.1)	53(54.6)	0.84	55(87.3)	53(86.9)	0.95	NA	NA	NA
Complete clinical remission, n (%)	24(24.5)	30(30.9)	0.32	24(38.1)	30(49.2)	0.21	NA	NA	NA
Daily defined doses	1.0(0,1.5)	0.3(0,1.5)	0.21	0(0,1.0)	0(0,0)	0.07	1.5(1.0,2.3)	1.5(0.9,2.3)	0.69
Adverse events, n (%)	40(40.8)	32(33.0)	0.26	31(49.2)	24(39.3)	0.27	9(25.7)	8(22.2)	0.73
Achieving target blood pressure, n (%)	66(67.3)	60(61.9)	0.42	44(69.8)	40(65.6)	0.61	22(62.9)	20(55.6)	0.53
SBP (mmHg)	128±14	130±17	0.49	128±14	128±19	0.85	129±14	132±14	0.36
DBP (mmHg)	85±11	87±13	0.33	85±11	87±14	0.40	85±10	86±11	0.63

* Patients with LI 2-4 were excluded (seventeen in non-ACTH and sixteen in ACTH stimulated group).

Data were expressed as %, median (interquartile range), or mean ± SD. SBP: systolic blood pressure; DBP: diastolic blood pressure; NA: not applicable. P values represent the comparison between non-ACTH stimulated and ACTH stimulated.

© 2023 Yang S et al. *JAMA Network Open*.

eTable 13. Outcomes at 12th month follow-up with increased selectivity index*

	Total (n=174)			Adrenalectomy(n=107)			Mineralocorticoid receptor antagonist(n=67)		
	non-ACTH stimulated (n=81)	ACTH stimulated (n=93)	P value	non-ACTH stimulated (n=52)	ACTH stimulated (n=55)	P value	non-ACTH stimulated (n=29)	ACTH stimulated (n=38)	P value
Complete biochemical remission, n (%)	44(54.3)	48(51.6)	0.72	44(84.6)	48(87.3)	0.69	NA	NA	NA
Complete clinical remission, n (%)	18(22.2)	28(30.1)	0.24	18(34.6)	28(50.9)	0.09	NA	NA	NA
Daily defined doses	1.0(0,1.6)	0.5(0,2.0)	0.54	0(0,1.0)	0(0,0)	0.15	1.6 (0.9,2.3)	1.8(0.8,2.3)	0.90
Adverse events, n (%)	34(42.0)	31(33.3)	0.24	28(53.8)	22(40.0)	0.15	6(20.7)	9(23.7)	0.77
Achieving target blood pressure, n (%)	57(70.4)	60(64.5)	0.41	36(69.2)	36(65.5)	0.68	21(72.4)	24(63.2)	0.42
SBP (mmHg)	129±13	130±17	0.58	128±13	128±17	0.78	129±12	133±17	0.27
DBP (mmHg)	85±10	87±12	0.35	85±10	87±13	0.45	85±10	87±12	0.59

*Increasing the AVS selectivity index from 3 to 5 in the ACTH stimulated group and from 2 to 3 in the non-ACTH stimulated group. Patients whose treatment did not comply with AVS results were excluded, including: 1) patients considered as indeterminant subtype or BPA by AVS (patients with SI <5 in ACTH stimulated group; patients in non-ACTH stimulated group with SI <3; SI ⩾5 in ACTH stimulated group plus LI <4; SI ⩾3 in non-ACTH stimulated group plus LI <4) but treated with adrenalectomy; 2) patients considered as UPA by AVS (SI ⩾5 in ACTH stimulated group plus LI ⩾4; SI ⩾3 in non-ACTH stimulated group plus LI ⩾4) but treated with medication.

Data were expressed as %, median (interquartile range), or mean ± SD. SBP: systolic blood pressure; DBP: diastolic blood pressure; NA: not applicable. P values represent the comparison between non-ACTH stimulated and ACTH stimulated.

© 2023 Yang S et al. *JAMA Network Open*.

eTable 14. Outcomes at 12th month follow-up with decreased LI*

	Total (n=168)			Adrenalectomy(n=124)			Mineralocorticoid receptor antagonist(n=44)		
	non-ACTH stimulated (n=83)	ACTH stimulated (n=85)	P value	non-ACTH stimulated (n=65)	ACTH stimulated (n=59)	P value	non-ACTH stimulated (n=18)	ACTH stimulated (n=26)	P value
Complete biochemical remission, n (%)	56(67.5)	51(60.0)	0.31	56(86.2)	51(86.4)	0.96	NA	NA	NA
Complete clinical remission, n (%)	24(28.9)	29(34.1)	0.47	24(36.9)	29(49.2)	0.17	NA	NA	NA
Daily defined doses	0.4(0,1.3)	0.1(0,1.5)	0.74	0(0,1.0)	0(0,0.2)	0.15	1.3(1.0,2.1)	1.9(0.7,2.4)	0.83
Adverse events, n (%)	36(43.4)	27(31.8)	0.12	31(47.7)	23(39.0)	0.33	5(27.8)	4(15.4)	0.32
Achieving target blood pressure, n (%)	57(68.7)	55(64.7)	0.59	45(69.2)	38(64.4)	0.57	12(66.7)	17(65.4)	0.93
SBP (mmHg)	127±14	129±16	0.41	127±14	128±16	0.78	128±13	132±15	0.34
DBP (mmHg)	85±11	87±12	0.42	85±11	87±13	0.39	85±10	86±11	0.89

***** Decreasing the AVS lateralization index from 4 to 2. Patients whose treatment did not comply with AVS results were excluded, including: 1) patients considered as indeterminant subtype or BPA by AVS (patients with SI <3 in ACTH stimulated group; patients in non-ACTH stimulated group with SI <2; SI ⩾3 in ACTH stimulated group plus LI <2; SI ⩾2 in non-ACTH stimulated group plus LI <2) but treated with adrenalectomy; 2) patients considered as UPA by AVS (SI ⩾3 in ACTH stimulated group plus LI ⩾2; SI ⩾2 in non-ACTH stimulated group plus LI ⩾2) but treated with medication. Data were expressed as %, median (interquartile range), or mean ± SD. SBP: systolic blood pressure; DBP: diastolic blood pressure; NA: not applicable. P values represent the comparison between non-ACTH stimulated and ACTH stimulated.

© 2023 Yang S et al. *JAMA Network Open*.

eReferences

1. Funder JW, Carey RM, Mantero F, et al. The Management of Primary Aldosteronism: Case Detection, Diagnosis, and Treatment: An Endocrine Society Clinical Practice Guideline. *J Clin Endocrinol Metab*. May 2016;101(5):1889-916. doi:10.1210/jc.2015-4061
2. Yanase T, Oki Y, Katabami T, et al. New diagnostic criteria of adrenal subclinical Cushing's syndrome: opinion from the Japan Endocrine Society. *Endocr J*. Apr 26 2018;65(4):383-393. doi:10.1507/endocrj.EJ17-0456
3. Song Y, Yang S, He W, et al. Confirmatory Tests for the Diagnosis of Primary Aldosteronism: A Prospective Diagnostic Accuracy Study. *Hypertension*. Jan 2018;71(1):118-124. doi:10.1161/HYPERTENSIONAHA.117.10197
4. Thuzar M, Young K, Ahmed AH, et al. Diagnosis of Primary Aldosteronism by Seated Saline Suppression Test-Variability Between Immunoassay and HPLC-MS/MS. *J Clin Endocrinol Metab*. Mar 1 2020;105(3)doi:10.1210/clinem/dgz150
5. Song Y, Yang J, Shen H, et al. Development and validation of model for sparing adrenal venous sampling in diagnosing unilateral primary aldosteronism. *J Hypertens*. Sep 1 2022;40(9):1692-1701. doi:10.1097/HJH.0000000000003197
6. Laurent I, Astere M, Zheng F, et al. Adrenal venous sampling with or without adrenocorticotropic hormone stimulation: A meta-analysis. *J Clin Endocrinol Metab*. Nov 6 2018;doi:10.1210/jc.2018-01324

© 2023 Yang S et al. *JAMA Network Open*.
