# Supplementary material for: Corticotropin Stimulation in Adrenal Venous Sampling for Patients With Primary Aldosteronism: The ADOPA Randomized Clinical Trial
Source: JAMA Netw Open. 2023 Oct 23;6(10):e2338209. doi: 10.1001/jamanetworkopen.2023.38209 (PMC10594148; doi:10.1001/jamanetworkopen.2023.38209)
Supplement: Supplement 3. — Nonauthor Collaborators. Chongqing Primary Aldosteronism Study (CONPASS) Group [file jamanetwopen-e2338209-s003.pdf]

Supplemental Online Content: Nonauthor Collaborators
*First name, last name, and suffix (if applicable) are required and will appear in PubMed.

*Group Name(s): Chongqing Primary Aldosteronism Study (CONPASS) Group							
***First Name and Middle Initial(s)**	***Last Name**	***Suffix (eg, Jr, III)**	Academic Degrees	Institution	Location (city, state/province, country)	Role or Contribution, eg, chair, principal investigator	Group (if more than 1 Group listed in the byline) and/or Subgroup (eg, Steering Committee)
Mei	Mei		MD, PhD	The First Affiliated Hospital of Chongqing Medical University	Chongqing,China	revision	
Suxin	Luo		MD, PhD	The First Affiliated Hospital of Chongqing Medical University	Chongqing,China	suggestions of study design	
Kangla	Liao		MD	The First Affiliated Hospital of Chongqing Medical University	Chongqing,China	suggestions of study design	
Yao	Zhang		MD, PhD	The First Affiliated Hospital of Chongqing Medical University	Chongqing,China	suggestions of study design	
Yunfeng	He		MD	The First Affiliated Hospital of Chongqing Medical University	Chongqing,China	revision	
Yihong	He		MD	The First Affiliated Hospital of Chongqing Medical University	Chongqing,China	revision	
Ming	Xiao		PhD	Chongqing Medical University	Chongqing,China	revision	
Bin	Peng		PhD	Chongqing Medical University	Chongqing,China	suggestions of study design	
