# Supplementary material for: Corticotropin Stimulation in Adrenal Venous Sampling for Patients With Primary Aldosteronism: The ADOPA Randomized Clinical Trial
Source: JAMA Netw Open. 2023 Oct 23;6(10):e2338209. doi: 10.1001/jamanetworkopen.2023.38209 (PMC10594148; doi:10.1001/jamanetworkopen.2023.38209)
Supplement: Supplement 4. — Data Sharing Statement [file jamanetwopen-e2338209-s004.pdf]

# Data Sharing Statement

Yang. Corticotropin Stimulation in Adrenal Venous Sampling for Patients With Primary Aldosteronism. *JAMA Netw Open*. Published October 23, 2023. doi:10.1001/jamanetworkopen.2023.38209

**Data**
**Data available:** No
